# Seven decades of chemotherapy clinical trials: a pan-cancer social network analysis

**DOI:** 10.1038/s41598-020-73466-6

**Published:** 2020-10-16

**Authors:** Xuanyi Li, Elizabeth A. Sigworth, Adrianne H. Wu, Jess Behrens, Shervin A. Etemad, Seema Nagpal, Ronald S. Go, Kristin Wuichet, Eddy J. Chen, Samuel M. Rubinstein, Neeta K. Venepalli, Benjamin F. Tillman, Andrew J. Cowan, Martin W. Schoen, Andrew Malty, John P. Greer, Hermina D. Fernandes, Ari Seifter, Qingxia Chen, Rozina A. Chowdhery, Sanjay R. Mohan, Summer B. Dewdney, Travis Osterman, Edward P. Ambinder, Elizabeth I. Buchbinder, Candice Schwartz, Ivy Abraham, Matthew J. Rioth, Naina Singh, Sanjai Sharma, Michael K. Gibson, Peter C. Yang, Jeremy L. Warner

**Affiliations:** 1 Vanderbilt University, Nashville, TN USA; 2 Mount Holyoke College, South Hadley, MA USA; 3 Stanford University, Palo Alto, CA USA; 4 Mayo Clinic, Rochester, MN USA; 5 Vanderbilt University Medical Center, 2220 Pierce Ave, PRB 777, Nashville, TN 37232 USA; 6 Massachusetts General Hospital, Harvard Medical School, Boston, MA USA; 7 University of Illinois at Chicago, Chicago, IL USA; 8 University of Washington, Seattle, WA USA; 9 Saint Louis University, St. Louis, MO USA; 10 University of North Dakota, Grand Forks, ND USA; 11 Rush University, Chicago, IL USA; 12 Mount Sinai Medical Center, New York, NY USA; 13 Dana-Farber Cancer Institute, Harvard Medical School, Boston, MA USA; 14 University of Colorado, Denver, CO USA; 15 Sequoia Regional Cancer Center, Visalia, CA USA

**Keywords:** Medical research, Clinical trial design, Randomized controlled trials, Cancer

## Abstract

Clinical trials establish the standard of cancer care, yet the evolution and characteristics of the social dynamics between the people conducting this work remain understudied. We performed a social network analysis of authors publishing chemotherapy-based prospective trials from 1946 to 2018 to understand how social influences, including the role of gender, have influenced the growth and development of this network, which has expanded exponentially from fewer than 50 authors in 1946 to 29,197 in 2018. While 99.4% of authors were directly or indirectly connected by 2018, our results indicate a tendency to predominantly connect with others in the same or similar fields, as well as an increasing disparity in author impact and number of connections. Scale-free effects were evident, with small numbers of individuals having disproportionate impact. Women were under-represented and likelier to have lower impact, shorter productive periods (*P* < 0.001 for both comparisons), less centrality, and a greater proportion of co-authors in their same subspecialty. The past 30 years were characterized by a trend towards increased authorship by women, with new author parity anticipated in 2032. The network of cancer clinical trialists is best characterized as strategic or mixed-motive, with cooperative and competitive elements influencing its appearance. Network effects such as low centrality, which may limit access to high-profile individuals, likely contribute to the observed disparities.

## Introduction

The modern era of chemotherapy began in 1946, with publications describing therapeutic uses of nitrogen mustard^1,2^. Over the next 70 years, the repertoire of available cancer treatments has expanded at an ever-increasing pace. Chemotherapeutics have a notably low therapeutic index, i.e., the difference between a harmful and beneficial dose or combination is often quite small^3^. Consequently, a complex international clinical trial apparatus emerged in the 1970s to study chemotherapeutics in controlled settings, and prospective clinical trials remain the gold standard by which standard of care treatments are established^4,5^. Discoveries made by successive generations have led to overall improvement in the prognosis of most cancers^6^.

While social network analysis has been used to study patterns of co-authorship in scientific settings^7,8^, the social component of clinical trial research is not well characterized. Little is known about how social factors have shaped the progress of the field, as cancer care has become increasingly subspecialized, and how social network characteristics may reveal patterns of inclusiveness, exclusivity, and disparity. Prior literature has established that women in academic medicine are expected to perform to higher standards yet receive less financial and institutional support (e.g., grants, awards, salaries, opportunities for tenure) than men with the same qualifications do^9–13^. Women must also contend with structural biases against them throughout their careers; this can manifest in gendered harassment and evaluations, questions of competence, lack of appropriate mentorship and peer support, and other inhospitable working conditions where they are undervalued, if not actively excluded^14^. Past work on co-authorship networks indicates that men are overrepresented in senior authorship roles, particularly in high-impact journals and in fields where research is more costly, and accrue more citations than their counterparts who are women^15–19^.

We hypothesized that the social network of cancer clinical trialists would be a “strategic” aka “mixed motive” network, similar to those found in other areas of academic research displaying both collaborative and competitive elements reflective of the pressures faced by clinial trialists; this type of social network may be especially prone to preferential attachment, where authors with many co-authorship links are more likely to form new links or strengthen existing ones than authors with few existing links^20^. This could contribute to a widening disparity between authors with early access to resources through personal networks and authors in more socially disadvantageous positions, e.g., those who come from low-income backgrounds or were the first in their family to attend college^21,22^. These groups are more likely to include underrepresented minorities, e.g., women of color, who face a number of discriminatory barriers in pursuing careers in medicine and medical research^11,23,24^.

In this study, we analyzed the individual impact and collaborative relationships of cancer clinical trialists, using co-authorship to define the edges, or links, of social networks. Our primary objective was to model the social network and its dynamic development over time; secondary objectives were to examine the roles of subspecialization and gender in relation to metrics of productivity, such as individual author impact, longevity in the field, position within the network, and similarity to co-authors (homophily).

## Results

### Baseline characteristics

N = 5599 of 6301 reviewed publications with an aggregate of N = 29,197 authors met the inclusion criteria (CONSORT Figure S1). Cumulatively, most authors in the network (n = 22,761, 78%) published at least one randomized trial, with n = 15,340 (52.5%) participating in the publication of a “positive” trial (Table S2). Most of the included authors (n = 28,087, 96.2%) participated in the primary publication of a clinical trial, while a smaller subgroup (n = 6,773, 23.2%) participated in the publication of updates. The most common venues for publication were high-impact clinical journals: the *Journal of Clinical Oncology* (n = 1595, 28.5%), the *Lancet* family (n = 710, 12.7%), the *New England Journal of Medicine* (n = 495, 8.8%), and the *Blood* family (n = 495, 8.8%). Co-authorship has changed in a non-linear fashion over time: the median number of authors per publication increased from n = 6 in 1946 to n = 20 (IQR 16–25) in 2018 (Figure S2). Across subspecialties, the median number of co-authors per publication varied somewhat, from a low of n = 10 (IQR 7–15) in gynecologic oncology to a high of n = 16 (IQR 11–22) in dermatologic oncology.

### Network dynamics

**Figure 1:**
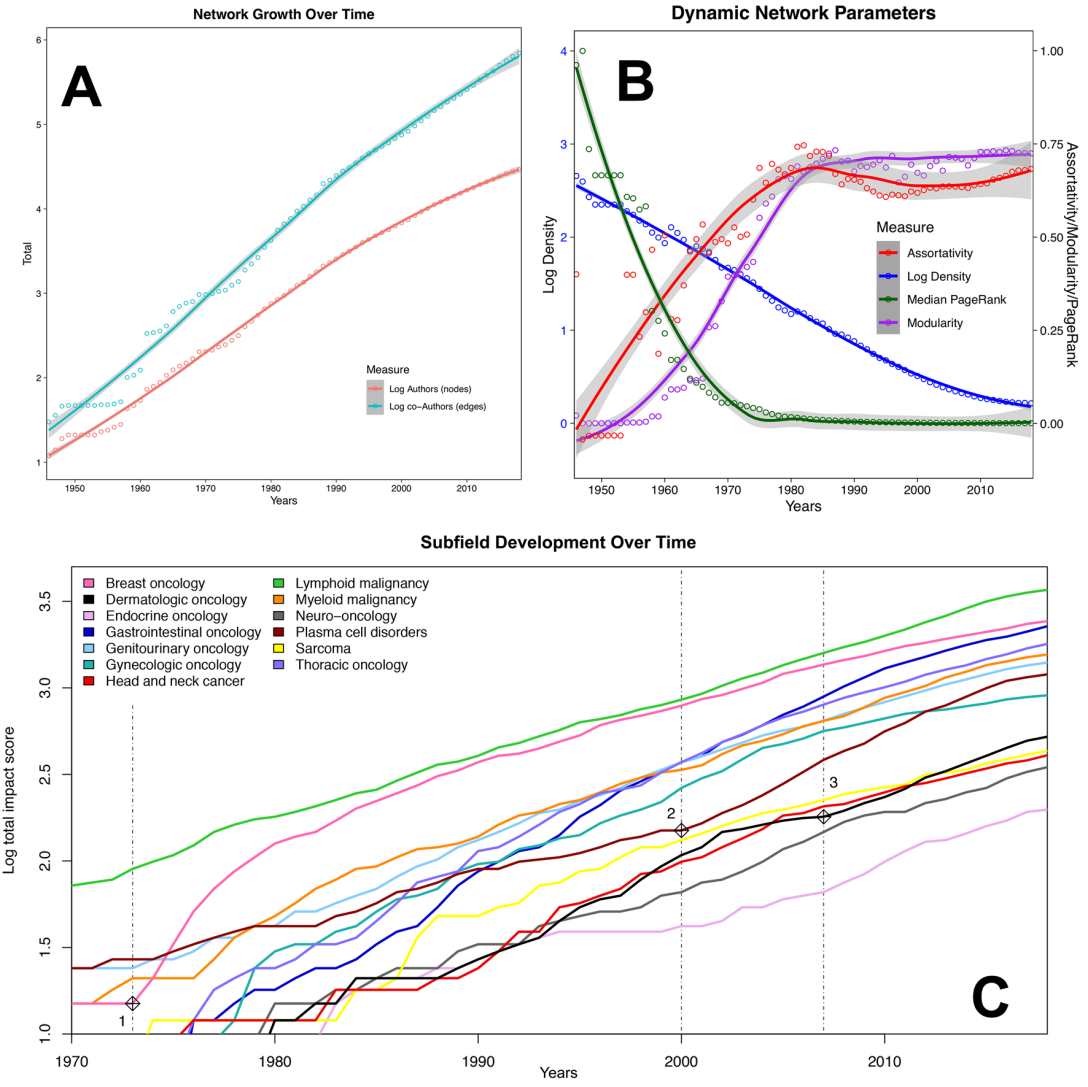
Network characteristics. (**A**) Cumulative growth in authorship and co-authorship over time have both been nearly log-linear; (**B**) Network density decreases asymptotically from 45.5% in 1946 to 0.16% in 2018; modularity follows a sigmoid pattern with a period of linear increase between 1960–80 followed by a plateau at high modularity; assortativity rapidly increases in early decades; median normalized PageRank decreases to a low plateau from the 1970s onward; (**C**) Subspecialties develop at different but broadly parallel rates, with seminal events apparently preceding accelerations of individual subspecialties, e.g.,: (1) in the four years after 1973, combination therapy (AC^25^), adjuvant therapy^26^, and tamoxifen^27^ were introduced in breast cancer; (2) thalidomide^28^ and bortezomib^29^ were reported to be efficacious for multiple myeloma; and (3) immunotherapy (ipilimumab^30,31^) was introduced in the treatment of melanoma.

Authorship and co-authorship have grown by over 200-fold: in 1946 there were 12 authors & 30 co-authors; by 2018, there were cumulatively 29,197 authors & 697,084 co-authors (Fig. 1A).

Median longevity is < 1 year at all times, although the number of authors with multiple years in the field grows substantially over time (Figure S3). A small number of individuals maintained the highest impact over time—nearly 20 years each in the case of chemotherapy pioneers Sidney Farber and James F. Holland (Figure S4). In any given year, most authors had a betweenness centrality of < 1% of the maximum; conversely, a very small number of authors had an exceptionally high score, with 1% of authors accounting for 100% of the total in recent years (Figure S5). Accordingly, an increasingly smaller proportion of authors were both very highly connected and highly impactful; in 1970, the 10% highest-impact authors (n = 20) account for 21.4% of links and 54.9% of impact; in 2018, the same proportion (n = 2920) account for 37.1% of links and 62.3% of impact. First/last authorship has also become concentrated; in 2018 publications, 10% of authors had at least one such role, whereas prior to 1980 it was on average > 25% (Figure S6).

The structure of the network changes considerably over time, from relatively dense and connected to sparse and modular (Fig. 1B). The final network is very sparse (0.16% of possible links are present); nevertheless, n = 29,029 (99.4%) authors are in a single connected component; the next-largest component comprises 14 authors. Each of the 13 cancer subspecialties developed at different rates, with clear influence of seminal events in several subspecialties, e.g., the introduction of adjuvant therapy and tamoxifen for breast cancer, completely new classes of drugs for plasma cell disorders, and immunotherapy for melanoma (Fig. 1C)^25–31^.

### Network visualization and cumulative metrics

**Figure 2:**
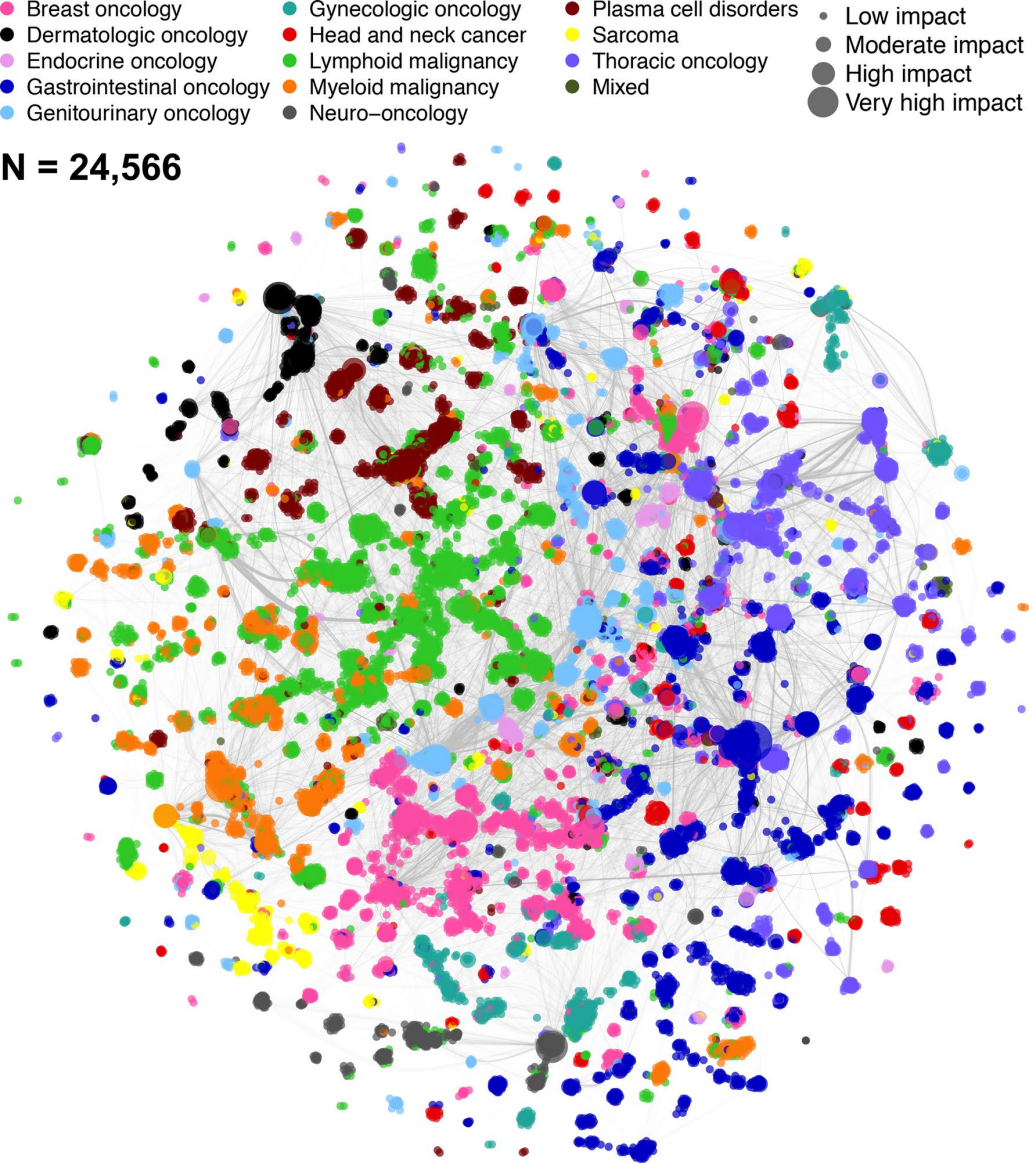
Final cumulative network visualization. The social network graph represents the cumulative field of cancer research as of December 31, 2018, with all included published works since 1946 contributing to authorship and co-authorship weights. Only authors assigned to a subspecialty are visualized; these account for 84% of all authors in the database. This figure highlights various clustering trends by subspecialty, such as the apparent sub-clusters of sarcoma research (yellow) and the two dominant clusters of breast cancer research (pink). It is clear as well that certain subspecialties are more cohesive than others, such as the tightly clustered dermatology (black) compared to the spread-out head and neck cancer authors (red).

The final cumulative network visualization is shown in Figs. 2 & S7. The impact score of authors is unevenly distributed, median 0.0532 (range 0–14.31); however, the log-transformed impact scores approximate a normal distribution (Figure S8). Authors with longevity ≥ 1 year who changed primary subspecialty at least once (n = 2330) had nearly twice the median impact and longevity of those who remained in one subspecialty (n = 10,276), 0.25 (IQR 0.11–0.6) versus 0.14 (IQR 0.07–0.35) and 13 years (IQR 6–19) versus 7 years (IQR 3–12), respectively (*P* < 0.001 for both comparisons).

Cumulatively, subspecialized authors with calculable homophily (n = 24,560) have a median proportion of co-authors sharing the same subspecialty of 88% (IQR 76–95%); 945,167 (71.4%) of these authors’ outlinks are within-subspecialty. This is reflected by a high assortativity by subspecialty since the mid-1960s (Fig. 1B).

### Gender disparities

**Figure 3:**
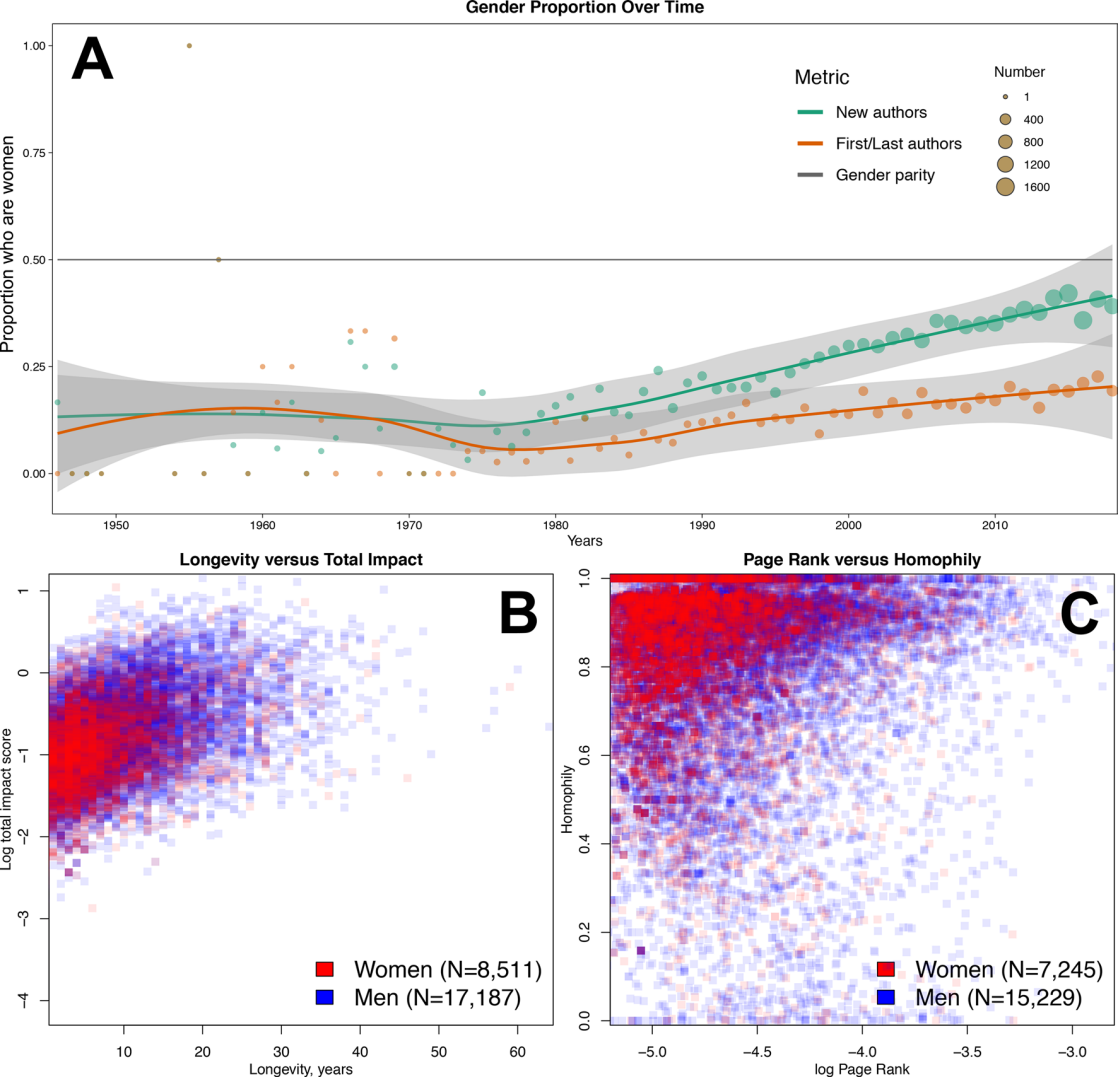
Gender disparities in the network. (**A**) The network is overwhelmingly dominated by men until 1980, when a trend towards increasing authorship by women begins to be seen; however, representation by women in first/last authorship remains low; gray shaded lines are 95% confidence intervals of the LOESS curves; (**B**) Men tend on average to have a longer productive period and to achieve a higher author impact score than women (*P* < 0.001 for both comparisons); (**C**) Men tend on average to be more central and have more collaborations outside of their subspecialty. Note that the homophily calculation requires a subspecialty assignment, which explains the slightly lower numbers in (**C**) as compared to (**B**).

**Figure 4:**
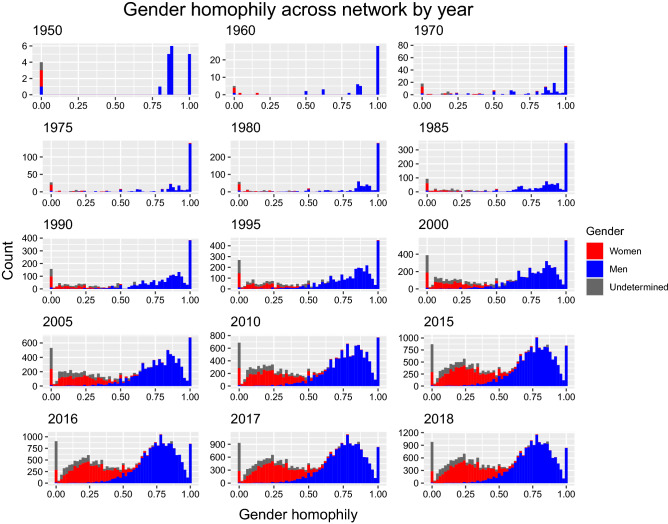
Gender homophily across the network by year. A value of one indicates co-authorship exclusively with co-authors of the same gender, whereas a value of zero indicates co-authorship exclusively with co-authors of another gender. Values among men trend higher than among women, indicating that men generally collaborate more with men, as do women. The y-axis range changes across the years to reflect the rapidly increasing size of the overall network.

The proportion of authors who are women remained at a nearly steady state of 15% until 1980, when it began to gradually increase to a recent high of ~ 40%; over the same time period, the proportion of women who were first/last authors rose much more slowly (Fig. 3A). Cumulatively, n = 17,187 men had a statistically significantly higher median impact than n = 8511 women, 0.075 (IQR 0.032–0.22) versus 0.051 (IQR 0.022–0.133), *P* < 0.001; statistically significantly longer median longevity, one year (IQR 0–8) versus zero years (IQR 0–5), *P* < 0.001; and higher median PageRank, 1.75 × 10^–5^ (IQR 1.01 × 10^–5^-3.68 × 10^–5^) versus 1.34 × 10^–5^ (IQR 8.74 × 10^–6^-2.51 × 10^–5^). For the n = 15,229 men and n = 7245 women with a calculable subspecialty homophily, men and women had comparable median proportions of co-authors within the same primary subspecialty, at 0.88 (IQR 0.75–0.95) and 0.89 (IQR 0.78–0.95) respectively. Gender homophily across the network over time shows that men have consistently been more likely to collaborate with those of the same gender than women; in the final network, median gender homophily among men was 0.77 (IQR 0.67–0.86) versus 0.26 (IQR 0.17–0.38) among women. Scatterplots of longevity versus author impact score and PageRank versus homophily are shown in Fig. 3B,C for the final cumulative network; prior years are shown in Figure S9 & S10. Histograms of gender homophily across time are shown in Fig. 4.

### Sensitivity analysis

Normalized score distributions did not change significantly, although modulation of the trial design coefficient led to a bimodal peak (Figure S11). Correlation of assortativity and modularity was high, ranging from 0.815–0.999 for the former and 0.981–0.999 for the latter (Table S3; Figure S12).

## Discussion

The remarkable gains in the fields of hematology and oncology can be ascribed to the tireless work of numerous trialists and the generosity of countless patient participants. As a result, systemic antineoplastics now stand beside surgery and radiotherapy as a pillar of cancer care. Our analysis of clinical trialists as a social network, particularly with respect to the density distribution of PageRank, reveals a mixed-motive network that differs substantially from “collegial” and “friend-based” online social networks. While clinical trials are conducted towards a collaborative goal—improved outcomes for all cancer patients—there are significant competitive pressures. Examples of these pressures include resource limitations (e.g., funding and patients available for accrual), the tension between prioritization of cooperative group versus industry-funded trials, personal motivations such as academic promotion or leadership opportunities, and institutional reputation.

The emergence of formal and informal leaders in scientific networks has been shown to facilitate research as well as create clusters^32^. As Fig. 2 shows, there is a strong tendency for clustering based on subspecialty in the complete network, although some subspecialties (e.g., lymphoid and myeloid malignancies) have many more interconnections than others (e.g., sarcoma and neuro-oncology). Many of these clusters appear to be organized around an individual or group of individuals who have high impact and centrality. As an organizational principle, these individuals appear to rarely be in direct competition, but their presence is a clear indicator of scale-free phenomena within the network. The facts that betweenness centrality follows a power law cumulative distribution bolsters this theory. Scale-free phenomena, which are defined by a power law distribution of connectedness, are very common in strategic networks, especially when they become increasingly sparse, as this network does^33^. The two related theories for this network behavior are preferential attachment and fitness. The former observes that those with impact tend to attract more impact; the latter postulates that such gains for the “fittest” come at the expense of the “less fit”^34^. Seminal events (Fig. 1C) are likely a driver of preferential attachment^35^, and may partially explain why some authors change their primary subspecialty at least once over time (e.g., through a “bandwagon” effect driven by the diffusion of ideas^36^). Given that these authors were observed to have nearly twice the impact and longevity of their single subspecialty peers, this dynamic will be a focus of future study, including calculation of the Q factor, a metric developed to quantify the ability of a scientist to “take advantage of the available knowledge in a way that enhances (Q > 1) or diminishes (Q < 1) the potential impact *p* of a paper”^37^.

In the analysis of network dynamics (Fig. 1B), the field as a whole appears to emerge in the 1970s, which is also when medical oncology and hematology were formally recognized through board certification. Measurements of field maturity are by their nature subjective, but the pessimism^38^ of the late 1960s was captured by Sidney Farber: “…the anticancer chemicals, hormones, and the antibiotics…marked the beginning of the era of chemotherapy of cancer, which may be described after 20 years as disappointing because progress has not been more rapid…”^39^. These concerns prompted the US National Cancer Act of 1971, which was followed by the leveling of modularity at a very high level from 1976 onwards, suggesting that the subspecialties generated in the 1970s have remained stable. The assortativity by subspecialty has increased as well, with recent levels approximately twice those seen in a co-authorship network of physicists^20^. While median PageRank has decreased markedly, indicating decreasing influence for the average author, the distribution in 2018 is broadly right-skewed (Figure S13). These findings reveal a high level of increasing exclusivity, suggesting that it is becoming progressively more difficult to join the top echelon of the network. This has major implications for junior investigators’ mobility, and potentially for the continued health of the network as a whole.

While there is much to be applauded in the continued success of translating research findings into the clinic, we observed clear gender disparities within the cancer clinical trialist network: women have a statistically significantly lower final impact score, shorter productive period, less centrality, and less collaboration with those outside of their primary subspecialty. These findings are consistent with and build upon previous literature on the challenges facing women in pursuing and remaining in academic careers^10,16,19,40^. They are also consistent with more recent gender disparity findings, such as those observed in research published on COVID-19^41^. Other studies investigating the basis for such a gender gap have identified several layers of barriers to the advancement of women in academic medicine. These include sexism in the academic environment, lack of mentorship, and inequity with regards to resource allocation, salary, space, and positions of influence^42,43^. Our study suggests that additional network factors such as relatively low centrality, which indicates a lack of access to other individuals of influence, and high homophily, which indicates a lack of access to new ideas and perspectives, also perpetuate the gender gap—corroborating recent findings from graduate school social networks^44^.

It is somewhat encouraging that there has been a steady increase in the proportion of authorship by women since 1980 (Fig. 3A). This increase is observed approximately a decade after the passage of Title IX of the US Civil Rights Act in 1972. Given that the majority of authors in this network are clinicians, a partial explanation could be that US-based women began to attend previously all-male medical schools in the early 1970s, completed their training, and began to contribute to the network as authors approximately 10 years later. If the nearly linear trend continues, we predict that gender parity for new authors entering the network will be reached by the year 2032, 26 years after US medical school enrollment approached parity^45^. However, the proportion of first/last authors who are women is growing much more slowly, and parity may not be reached for 50+ years, if at all. Given that senior authorship is a traditional metric of scholarly productivity, it may be particularly difficult for clinical trialists who are women to obtain promotion under the current paradigm. One possible solution is to increase the role of joint senior authorship, which remains vanishingly rare in the clinical trials domain (Furman et al. 2014^46^ is one of very few examples that we are aware of)—although this is predicated on the acceptance of these roles by advancement and promotion committees. The field itself may also suffer from slow entry of new talent and a lack of broad perspectives.

While the gender mapping algorithm and manual lookups are imperfect, our approach is consistent with prior work in this area^16,47^. Unisex names posed a particular challenge^48^. It should be noted that we could not account for all situations where an author changed their name (e.g., a person assumed their spouse’s surname); this could have led to overestimation of representation by women and underestimation of impact, since this practice is more common with women. It is also possible that an individual’s gender identity does not match the gender assignment of their given name. Future work will include further analysis of gender disparities, factoring in institutional affiliation and highest degree(s) obtained, which are both likely to have significant influence on publication and senior authorship^49,50^.

There are several additional limitations to this work, starting with the fact that co-authorship is but one way to measure social network interactions and this study reports results from published trials, which induces publication bias. Although HemOnc.org aims to be the most comprehensive resource of its kind, non-randomized trials and randomized phase II trials are intentionally underrepresented, given that findings at this stage of investigation infrequently translate to practice-changing results (e.g., approximately 70% of oncology drugs fail during phase II)^51–53^. The effect of any biases introduced by this underrepresentation is unclear, given the confounding influence of publication bias, which may itself be subject to gender disparity^54^. Some older literature which no longer has practice-changing implications may have been overlooked.

During name disambiguation, some names could not be resolved, primarily because neither MEDLINE nor the primary journal site contained full names. This effect is non-random, since certain journals do not publish full names. The choice of coefficients and their relative weights was based on clinical intuition and consensus; given that the “worth” of metrics such as first/last authorship is fundamentally qualitative, there must be some degree of subjectivity when formulating a quantitative algorithm. While the sensitivity analysis demonstrated that neither normalized author impact score distribution, assortativity, nor modularity are majorly changed by variation in the trial design and author role coefficients, it remains possible that other combinations of coefficients and relative weightings could lead to different results. Furthermore, our impact algorithm weighs heavily on first and last authorship, but the definition of senior authorship has changed over time. For example, in the 1946 article by Goodman et al.^2^, the authors were listed in decreasing order of seniority (personal communication). In general, the impact score used in this paper, although similar to others proposed in the academic literature, is not validated and should be interpreted with caution. Finally, the majority of authors in this database publish extensively, and their impact as measured here should not be misconstrued to reflect their contributions to the cancer field more broadly.

In conclusion, we have described the first and most comprehensive social network analysis of the clinical trialists involved in chemotherapy trials. We found emergent properties of a strategic network and clear indications of gender disparities, albeit with improvement in representation in recent decades. The network has been highly modular and assortative for the past 40 years, with little collaboration across most subspecialties. As the field pivots from an anatomy-based to a precision oncology paradigm, it remains to be seen how the network will re-organize so that the incredible progress seen to date can continue.

## Methods

### Data source and curation process

All prospective trials of systemic antineoplastics published between 1946–2018 and referenced on HemOnc.org were considered for inclusion. HemOnc.org is the largest collaborative wiki of chemotherapy drugs and regimens and has a formal curation process^55^. In order for a reference to be included on HemOnc.org, it generally must include at least one regimen meeting the criteria outlined here: https://hemonc.org/wiki/Eligibility_criteria. As such, the majority of references on HemOnc.org are randomized controlled trials (RCTs) or non-randomized trials with at least 20 participants and/or practice-changing implications. One of the main goals of HemOnc.org is creating a database of all standard of care systemic antineoplastic therapy regimens. This is difficult as there is no universally accepted definition of standard of care except in a legal capacity. For example, the state of Washington, in its legislation on medical negligence, inversely defines the standard of care as “exercis[ing] that degree of skill, care, and learning possessed at that time by other persons in the same profession”. We currently employ four separate definitions that meet the threshold of standard of care:

1. The control arm of a phase III randomized controlled trial (RCT). By implication, this means that all phase III RCTs with a control arm must eventually be included on the website.
2. The experimental arm(s) of a phase III RCT that provide(s) reasonable evidence (P-value less than 0.10) of superior efficacy for an intermediate surrogate endpoint (e.g., PFS) or a strong endpoint (e.g., OS).
3. A non-randomized study that is either:
  (i) The basis for a regulatory agency approval (e.g., the US Food and Drug Administration [FDA])
  (ii) Recommended as a top-level regimen by the American Society of Clinical Oncology (ASCO), the European Society for Medical Oncology (ESMO), or the National Comprehensive Cancer Network (NCCN).
4. Any study (including case series and retrospective studies) that is specifically recommended by a member of the HemOnc.org Editorial Board. All section editors of the Editorial Board with direct oversight of disease-specific pages are board-eligible or board-certified physicians.

In order to identify new regimens and study references for inclusion on HemOnc.org, we undertake several parallel screening methods:

1. PubMed search for (“Phase 3”[Title/Abstract] OR “Phase III”[Title/Abstract]) AND “neoplasms”[MeSH Terms] AND Clinical Trial[ptyp] (annual; 2019 review currently underway)
2. PubMed email alert for “Clinical Trial, Phase III”[Publication Type] AND “Neoplasms”[Mesh] (subscription; ongoing per PubMed alerting criteria)
3. Review of the eTOC of the following “top-tier” general medical and hematology/oncology journals (subscription; ongoing per journal publication schedules):
  (i) JAMA
  (ii) The Lancet
  (iii) The New England Journal of Medicine
  (iv) Annals of Oncology
  (v) Blood
  (vi) JAMA Oncology
  (vii) Journal of Clinical Oncology
  (viii) The Lancet Haematology
  (ix) The Lancet Oncology
4. Time permitting, review of the eTOC of other hematology/oncology journals, conference proceedings, and e-mail alerts
5. Review of all freely available Cochrane Library Reviews under the topics “Cancer” and “Blood disorders” (biennial; last completed: October 2018)
6. Review of all ASCO, ESMO, and NCCN clinical practice guidelines (biennial; last completed: December 2018)
7. Review of all studies cited on the FDA drug label (“package insert”) section 14 (CLINICAL STUDIES) for all antineoplastic agents (ongoing for new approvals and new indications; review of all available existing labels completed: September 2019)
8. Queries to all Editorial Board members (quarterly).

As part of the process of building HemOnc.org, we have also systematically reviewed all *Lancet*, *JAMA*, and *New England Journal of Medicine* tables of contents from 1946 to December 31, 2018. In addition, the citations of any included manuscript are hand-searched for additional citations. For any treatment regimen that has been subject to randomized comparison, we additionally seek to identify the first instance in which such a regimen was evaluated as an experimental arm; if no such determination can be made, we seek the earliest non-randomized description of the regimen for inclusion on the website. In order or prioritization, phase III RCTs are added first, then smaller RCTs such as randomized phase II, followed by non-randomized trials, followed by retrospective studies or case series identified by our editorial board as relevant to the practice of hematology/oncology.

When a reference is added to HemOnc.org, bibliographic information including authorship is recorded. The usually coincides with MEDLINE record details, although some older references in MEDLINE are capped at ten authors and are manually completed based upon the publication of record. For trials that do not list individual authors (e.g., The Elderly Lung Cancer Vinorelbine Italian Study Group^56^), the original manuscript and appendices are examined for a writing committee. If a writing committee is identified, the members of this committee are listed as authors in the order that they appeared in the manuscript. If no writing committee is identified, the chairperson(s) of the study group are listed as the first & last authors. If no chairpersons are listed, the corresponding author is listed as the sole author.

Publications solely consisting of the evaluation of drugs not yet approved by the FDA or other international approval bodies were not included. Trials that appeared in abstract form only, reviews, retrospective studies, meta-analyses, and case reports were excluded, as were trials reporting only on local interventions such as surgery, radiation therapy, and intralesional therapy. Non-antineoplastic trials (Table S1) and trials of supportive interventions (e.g., antiemesis; growth factor support) were also excluded.

### Disambiguation of author names

For each included publication, author names were extracted and disambiguated. Author names on HemOnc.org are stored in the MEDLINE LastName_FirstInitial (MiddleInitial) format, which can lead to two forms of ambiguity: (1) the short form, e.g., *Smith_J*, can refer to two or more individuals, e.g., Julian and Jane Smith; (2) two short forms can refer to the same individual, e.g., *Kantarjian_H* and *Kantarjian_HM*. Additionally, names can be misspelled and individuals can change their name over time (e.g., a person assumes their spouse’s surname). We undertook several steps to disambiguate names: (1) full first and middle names, when available, were programmatically accessed through the NCBI PubMed eUtils^57^ application programming interface; (2) when not available through MEDLINE, full first names were searched for on journal websites or through web search engines; (3) automatic rules were developed to merge likely duplicates; and (4) some names were manually merged (e.g., misspellings: *Benboubker_Lofti* and *Benboubker_Lotfi;* alternate forms: *Rigal-Huguet_Francoise* and *Huguet-Rigal_Francoise*; and subsumptions: *Baldotto_Clarissa* and *Serodio da Rocha Baldotto_Clarissa*). Transformation algorithms are available upon request, and the full mapping table is provided in Supplemental File 1.

### Gender mapping

Once the name disambiguation step was complete, we mapped authors with full name available to gender. We first mapped names to genders using US census data, which includes the relative frequencies of given names by gender in the population of US birth from 1880 to 2017. We calculated the gender ratio for names that appeared as both genders. For names with gender ratio > 0.9 for one gender (e.g., John, Rebecca), we assigned the name to that gender. To expand gender mapping to include names that are more frequently seen internationally (e.g., Jean, Andreas), we used a program that searches from a dictionary containing gender information about names from most European countries as well as some Asian and Middle Eastern countries^58^. For unmatched first names (e.g., Dana, Michele), we manually reviewed for potential gender assignment. For some names that are masculine in certain countries and feminine in others (e.g., Andrea, Daniele, and Pascale are masculine in Italy and feminine elsewhere), we mapped based on surnames. Finally, we performed manual internet searches to look for photographs and pronouns used in online content such as faculty profiles, book biographies, and professional social media accounts for the remaining unmapped full names associated with a longevity of greater than one year.

A total of 25,698 (88%) authors were assigned to the categories of woman (N = 8511; 29.2%) or man (N = 17,187; 58.9%). The gender of most of the people with unassigned names could not be determined because they only appeared with initials (N = 2716; 9.3%) in the primary publication and MEDLINE. The remaining N = 685 (2.3%) were ambiguously gendered names that could not be resolved through manual searching, and were excluded in the gender-specific analyses. The full mapping table is provided in Supplemental File 2.

### Author impact score

We considered existing metrics for measuring author impact^59–62^, but ultimately proceeded with our own formulation given some of the unique considerations of prospective clinical trials and their impact. Every author was assigned an impact score, using an algorithm calculated per manuscript using four coefficients: (1) author role; (2) trial type; (3) citation score; (4) primary versus updated analysis. The coefficients are multiplied to arrive at the score, and the total author impact score is summed across all of their published manuscripts.

*Author Role:* First and last author roles are assigned a coefficient of three; middle authors are assigned a coefficient of one. When joint authorship is denoted in a MEDLINE record, there is an additional attribute “EqualContrib” that is set to “Y” (yes). We look for this during the parsing process and treat these authors as first or last authors when the attribute is detected.

*Trial Type:* Any prospective trial with randomization is denoted as randomized and the authors of any manuscript reporting on such a trial are assigned a coefficient of two. Non-randomized trials are assigned a coefficient of one. For manuscripts that reported on more than one trial with mixed designs (i.e., one or more randomized and one or more non-randomized trials), the randomized coefficient was used.

*Citation Score:* We programmatically obtained a snapshot of citation counts from Google Scholar from September 2019 and used unadjusted total citations as the citation score coefficient for the years 1946–2008. As more recent publications are still accruing citations, raw citation count is not an appropriate measure of their impact. Therefore, we have calculated a blended citation score for articles published between 2009–2018, adding the phased in median citation count for the journal tier in which the article was published for the years 1946–2008 (see Tables S4 & S5 and Figure S14). The citations scores are normalized to the manuscript with the maximum number of citations (Stupp et al. 2005^63^, with 13,341 citations), such that the maximum citation score is one.

*Primary Publications vs. Updates:* The baseline coefficient is one. For updates, this score is multiplied by a half-life decay coefficient; i.e., scores for the first update are multiplied by 50%; scores for the second update by 25%; and so forth. This rule is applied equally to updates and subgroup analyses. For manuscripts that reported on pooled updates of more than one trial, the score was multiplied by the half-life coefficient corresponding to the update that resulted in the maximum score.

See examples in Supplemental Methods.

### Subspecialty designation of each publication

Each publication was assigned to one of 13 disease-specific cancer subspecialties based on the cancer(s) studied (Table S1). The majority of publications report on a clinical trial carried out in one disease or several diseases mapping to the same subspecialty. For publications studying diseases that map to more than one subspecialty, each author’s impact score for that publication was divided evenly across the subspecialties. Several clinical trials employ a site-agnostic approach, e.g., to a “cancer of unknown primary” or to biomarker-defined subsets of cancers (e.g., a basket trial^64^); for these, impact across subspecialties was split manually (Table S6).

### Subspecialty designation based on authorship

Authors were eligible for assignment to a primary subspecialty based on whether they were a first or last author at least once in the subspecialty, or whether they had a cumulative impact of at least one standard deviation below the mean of the author impact score of all authors in the subspecialty. Authors who met either of these criteria were assigned to a primary subspecialty based on where the majority of their impact lay; if an author had equal impact in two or more subspecialties they were assigned equally to the subspecialties. This assignment was recalculated on an annual basis if the author had new publications, and primary subspecialty was re-assigned if a new subspecialty met either of the criteria and the impact in that subspecialty was higher than in the previous primary subspecialty. Authors not meeting either of these criteria were assigned a primary subspecialty of “None” and were not included in the homophily analysis or the network visualization.

### Author longevity

Author longevity was calculated as the interval between their first and final publication in the database. Given that preparing and publishing results of clinical trials can take substantial time, authors with first publications prior to 2016 and final ones in 2016 or 2017 had their final publication year adjusted to 2018.

### Social network construction and metrics

A dynamic social network was created with nodes representing authors and links representing co-authorship. The dynamic social network was discretized by year and the authors, scores, and links were cumulative (e.g., the 20^th^ network was cumulative from 1946–1965). Therefore, once an author is added to the network, they remain in the network, with their impact score cumulatively increasing as they publish and remaining constant if publication activity ceases. The following temporal metrics were calculated: (1) network density (the number of actual connections/links present divided by the total number of potential connections); (2) modularity^65^ by subspecialty (a measure of how strongly a network is divided into distinct communities, in this case subspecialties, defined as the number of edges that fall within a set of specified communities minus the number expected in a network with the same number of vertices and edges whose edges were placed randomly); (3) assortativity^66^ by subspecialty (a measure of the preference of nodes in a network to attach to others that are similar in a defined way, in this case the same subspecialty; assortativity is positive if similar vertices tend to connect to each other, and negative if they tend to not connect to each other); (4) betweenness centrality^67^ (a measure reflecting how important an author is in connecting other authors, calculated as the proportion of times that an author is a member of the bridge that forms the shortest path between any two other authors); (5) PageRank^68^ (another measure of centrality, this time considering the connection patterns among each author’s immediate neighbors; its value for each author is the probability that a person starting at any random author and randomly selecting links to other authors will arrive at the author); and (6) proportion of co-authors sharing either the same primary subspecialty designation or the same gender (hereafter referred to as homophily). Network density, modularity, and assortativity are calculated at the network level, while betweenness centrality, PageRank, and homophily are calculated at the author (node) level. Further definitions of these metrics are provided in the Supplemental Glossary.

All metrics incorporated the weighted co-authorship score, which takes into account each co-author’s impact modified by the number of authors of an individual publication. For each pairwise collaboration, as defined by co-authorship on the same manuscript, a co-authorship score was calculated and used as the edge weight; duplicated edges were allowed to reflect the fact that weights could be distributed in a non-even fashion (e.g., two co-authors could be middle authors on a lower-impact publication as well as senior authors on a separate high-impact publication). This score was first calculated by multiplying the individual authors’ manuscript-specific impact scores together. In order to acknowledge the role of middle authors in large multi-institutional studies, this preliminary score was divided by the total number of authors on the manuscript. This has the effect of decreasing the weight of any individual co-authorship relationship in a paper with many authors, while allowing the overall weight of the neighborhood consisting of all co-authorship connections to increase linearly with the number of authors (see examples in Supplemental Methods).

In order to visualize the final cumulative network, layout was determined using the distributed recursive graph algorithm^69^. Nodes were sized by author impact score rank and colored by primary subspecialty designation. Edge width was determined by the weighted co-authorship score.

### Statistical analysis

Non-independent network metrics including growth, density, assortativity, modularity, and PageRank are reported descriptively with medians and interquartile ranges (IQR). Gender proportion over time was fit with locally estimated scatterplot smoothing (LOESS) regression using default settings of degree = 2 with smoothing parameter/span α = 0.75^70^. For the final cumulative network, the independent variables author impact score and longevity were compared (1) between genders and (2) by whether the author changed subspecialties over time; only those authors with longevity ≥ 1 year were included in the second comparison. These comparisons were made with the two-sided Wilcoxon rank sum test; *P* value < 0.05 was considered statistically significant.

### Sensitivity analysis

To determine whether the scoring algorithm was robust to modifications, we conducted a sensitivity analysis where the author role and trial design coefficients were varied by ± 67% and ± 50%, respectively. Normalized density distributions for the final cumulative network under each permutation were calculated, and temporal assortativity and modularity were compared to baseline with Pearson’s correlation coefficient.

### Conference presentation

A version of this manuscript is posted on the medRxiv preprint server, accessible here: https://www.medrxiv.org/content/10.1101/19010603v1. A very early version of the work was presented in poster format at the 2018 Visual Analytics in Healthcare workshop (November 2018). There are no other prior presentations.

## Supplementary information

Supplementary Information 1.

Supplementary Information 2.

Supplementary Information 3.

## Data Availability

The datasets generated and analyzed in this study are available at Harvard Dataverse^71^.
