# Supplementary material for: Seven decades of chemotherapy clinical trials: a pan-cancer social network analysis
Source: Sci Rep. 2020 Oct 16;10:17536. doi: 10.1038/s41598-020-73466-6 (PMC7568560; doi:10.1038/s41598-020-73466-6)
Supplement: Supplementary file 1 — Supplementary Information 1. [file 41598_2020_73466_MOESM1_ESM.docx]

**Supplementary Material for: Seven Decades of Chemotherapy Clinical Trials: A Pan-Cancer Social Network Analysis**

Xuanyi Li, BS^1#^; Elizabeth A. Sigworth, BA^1#^; Adrianne H. Wu^2#^; Jess Behrens, MS^1^; Shervin A. Etemad, BS^1^; Seema Nagpal, MD^3^; Ronald S. Go, MD^4^; Kristin Wuichet, PhD^5^; Eddy J. Chen, MD^6^; Samuel M. Rubinstein, MD^5^; Neeta K. Venepalli, MD, MBA^7^; Benjamin F. Tillman, MD^5^; Andrew J. Cowan, MD^8^; Martin W. Schoen, MD, MPH^9^; Andrew Malty^3^; John P. Greer, MD^5^; Hermina D. Fernandes, MD, FACP^10^; Ari Seifter, MD^7^; Qingxia Chen, PhD^1^; Rozina A. Chowdhery, MD^7^; Sanjay R. Mohan, MD, MSCI^5^; Summer B. Dewdney, MD^11^; Travis Osterman, DO, MS^5^; Edward P. Ambinder, MD, FASCO^12^; Elizabeth I. Buchbinder, MD^13^; Candice Schwartz, MD^7^; Ivy Abraham, MD^7^; Matthew J. Rioth, MD, MS^14^; Naina Singh, MD^7^; Sanjai Sharma, MD^15^; Michael Gibson, MD, PhD^5^; Peter C. Yang, MD^6#^; and Jeremy L. Warner, MD, MS, FAMIA^5#*^

^1^Vanderbilt University, Nashville, TN, USA

^2^Mount Holyoke College, South Hadley, MA, USA

^3^Stanford University, Palo Alto, CA, USA

^4^Mayo Clinic, Rochester, MN, USA

^5^Vanderbilt University Medical Center, Nashville, TN, USA

^6^Massachusetts General Hospital, Harvard Medical School, Boston, MA, USA

^7^University of Illinois at Chicago, Chicago, IL, USA

^8^University of Washington, Seattle, WA, USA

^9^Saint Louis University, St. Louis, MO, USA

^10^University of North Dakota, Grand Forks, ND, USA

^11^Rush University, Chicago, IL, USA

^12^Mount Sinai Medical Center, New York, NY, USA

^13^Dana-Farber Cancer Institute, Harvard Medical School, Boston, MA, USA

^14^University of Colorado, Denver, CO, USA

^15^Sequoia Regional Cancer Center, Visalia, CA, USA

^#^contributed equally

**Research Support:** This work was supported by the Vanderbilt Initiative for Interdisciplinary Research (J.B.); NIH grants P30 CA068485 (J.L.W.), T32 CA009515 (A.J.C.), T32 HG008341 (S.M.R.), U01 CA231840 (K.W. and J.L.W.), U24 CA194215 (E.A.S. and Q.C.); and NSF grant #1757644 (A.H.W.).

***Corresponding author:** 2220 Pierce Ave, PRB 777, Nashville, TN 37232, USA. Ph 1-615-322-5464; [jeremy.warner@vumc.org](mailto:jeremy.warner@vumc.org)

**Running Head:** Pan-Cancer Social Network Analysis

**Prior Presentation/Publication:** A version of this manuscript is posted on the medRxiv preprint server, accessible here: <https://www.medrxiv.org/content/10.1101/19010603v1>. A very early version of the work was presented in poster format at the 2018 Visual Analytics in Healthcare workshop (November 2018). There are no other prior presentations.

# Table of Contents

[Glossary 4](#_Toc47956535)

[Supplemental Methods 6](#_Toc47956536)

[Author impact score calculation – examples 6](#_Toc47956537)

[Co-authorship score for edge weighting – examples 6](#_Toc47956538)

[Supplemental Figures 7](#_Toc47956539)

[Figure S1. CONSORT diagram of author eligibility selection process 7](#_Toc47956540)

[Figure S2. Number of authors per publication over time 8](#_Toc47956541)

[Figure S3. Author longevity over time 9](#_Toc47956542)

[Figure S4. Dominant authors’ share of the field over time 10](#_Toc47956543)

[Figure S5. Betweenness centrality (BC) over time 11](#_Toc47956544)

[Figure S6. Proportion of first/last authorship over time 12](#_Toc47956545)

[Figure S7. Final network visualization, colored by gender. 13](#_Toc47956546)

[Figure S8. Density distribution of author impact, 2018 14](#_Toc47956547)

[Figure S9. Total impact versus longevity, 1946-2017 15](#_Toc47956548)

[Figure S10. Homophily versus PageRank, 1946-2017 16](#_Toc47956549)

[Figure S11. Normalized author score distributions, sensitivity analysis 17](#_Toc47956550)

[Figure S12. Assortativity and modularity, sensitivity analysis 18](#_Toc47956551)

[Figure S13. Density distribution of PageRank scores, 2018 19](#_Toc47956552)

[Figure S14. Article citation score over time 20](#_Toc47956553)

[Supplemental Tables 21](#_Toc47956554)

[Table S1. Mapping of HemOnc.org cancer subtype and site-agnostic pages to subspecialties 21](#_Toc47956555)

[Table S2. Study characteristics for the final cumulative network 26](#_Toc47956556)

[Table S3. Sensitivity analysis results 28](#_Toc47956557)

[Table S4. Journal tiers of the 195 journals with publications in the analyzed database 29](#_Toc47956558)

[Table S5. Adjusted citation score for manuscripts published between 2009-2018 35](#_Toc47956559)

[Table S6. Re-mapping of site-agnostic references 36](#_Toc47956560)

[Supplemental Bibliography 37](#_Toc47956561)

# **Glossary**

**Vertex (aka node)**: one of the two fundamental units of a graph (together with edges), representing an object or concept, in this case individual authors. Vertices can have attributes assigned to them, for example author impact, specialty, or gender.

**Edge (aka link):** one of the two fundamental units of a graph (together with vertices), representing a relationship between two vertices, which in our case is a co-authorship connection. Each edge has two endpoints, the vertices to which they are attached.

**Dyad:** any two nodes which are connected by an edge form a dyad.

**Graph (aka network):** a collection of vertices and edges in which each edge represents a relationship, e.g., an edge between authors A and B represents a dyadic co-authorship relationship between them.

**Weighted Graph:** a graph where a characteristic beyond the presence or absence of an edge is used to calculate metrics such as assortativity and betweenness centrality. For example, a first-last author dyad is weighted differently than a middle author-middle author dyad in our network.

**Immediate neighbors:** two vertices which are connected by an edge. Two authors are immediate neighbors if they were co-authors on at least one paper. The collection of all immediate neighbors to a given vertex is called its neighborhood.

**Outlinks:** for each vertex, the set of edges that begin at that vertex and end at another vertex. For an author, outlinks are their set of co-authorship relationships.

**Degree:** the number of immediate neighbors for a given vertex, which is the sum of all outlinks.

**Component:** if a network is not fully connected (i.e., there is a way to trace a path from any vertex to any other vertex), then it is said to be divided into components. Each of the individual components in a network is fully connected.

**Social network:** a network of individuals, in this case authors, connected by some form of interpersonal relationship, in this case by co-authorship on published papers.

**Mixed motive network (aka strategic network**^1^**):** a network in which individuals (vertices) actively foster relationships (edges) that are beneficial to their success, and drop or cease to build upon relationships that are not.

**Dynamic social network:** a social network in which the vertices and edges between them can change over time. These changes can include attributes such as strength of a connection as well as addition and deletion of vertices and edges.

**Social network analysis:** the practice of investigating social structures via networks and graph theory.

**Network density:** the number of actual connections (links) present divided by the number of potential connections. Let *d* be the density of the network, *E* be the number of links, and *n* be the total number of nodes, then

$$d= \frac{E}{\frac{n(n-1)}{2}}.$$

**Modularity**^2^**:** a measure of how strongly a network is divided into distinct communities, defined as the number of edges that fall within a set of specified communities minus the number expected in a network with the same number of vertices and edges whose edges were placed randomly. A highly modular network has most of its edges between members that belong to the same communities (e.g. cancer subspecialties; social groups) and few between members that belong to different ones.

Define $e_{ab}$ to be the fraction of edges where one end lives in community *a* and the other in community *b*, let $A_{ij}$ be the weight of the edge connecting nodes *i* and *j*, and let $I\left( i\in c_{a} \right)$ be an indicator that node *i* lives in community *a*, then

$$e_{ab}=\sum_{ij} \frac{A_{ij}}{2m}I\left( i\in c_{a} \right)I(j\in c_{b}).$$

Next define $d_{a}$ to be the fraction of all edges that are attached to nodes in community *a* as

$$d_{a}= \frac{k_{a}}{2m}=\sum_{b} e_{ab}$$

where $k_{a}$ is the total degree of community *a*, which has *m* total edges. Then finally, the modularity *Q* for a network with *c* total distinct communities is

$$Q= \sum_{a=1}^{c} (e_{aa}-d_{a}^{2}).$$

**Homophily:** the generic tendency for individuals to form connections with people who are similar to them in some way.

**Assortativity**^3^: a specific preference for a network’s nodes to attach to others that are similar in a defined way. The assortativity coefficient is positive if similar vertices (based on some external property) tend to connect to each, and negative otherwise.

Define *r* to be the assortativity value,

$$r= \frac{\sum_{jk} jk(e_{jk}-q_{j}q_{k})}{\sigma_{q}^{2}}$$

where *j* and *k* are the excess degree of each node of interest (the degree of the node minus one), and $q_{k}$ captures the number of edges leaving node *k* other than the one that connects the pair, based on the degree distribution $p_{k}$ and written as

$$q_{k}= \frac{\left( k+1 \right)p_{k+1}}{\sum_{j\geq1} jp_{j}}$$

and let $e_{jk}$ be the joint excess degree probability for excess degree values *j* and *k* and $\sigma_{q}$ is the standard deviation of $q_{k}$.

**Centrality**: a generic measure for each node (author) ranking their relative importance within the network based on their relationships to others.

**Betweenness centrality**^4^**:** a measure of centrality where the proportion of times that an individual node (author) is a member of the bridge forming the shortest path between any two other nodes, reflecting how important the author is in connecting other authors, is enumerated. In a weighted graph, the weights are used to calculate the shortest paths, and are interpreted as distances. Given that a high weight in our network corresponds to a strong connection, we use the reciprocal of weight in our betweenness centrality calculation.

We can write this as

$$C_{B}\left( v \right)= \sum_{s\neq v\neq t\in V} \frac{\sigma_{st}(v)}{\sigma_{st}}$$

where $C_{B}\left( v \right)$ is the betweenness centrality value of node *v*, calculated across pairs of nodes *s* and *t* where $\sigma_{st}(v)$ is the number of shortest paths between *s* and *t* that involved node *v* and $\sigma_{st}$ is the total number of shortest paths from *s* to *t*, in a network of *V* nodes.

**PageRank**^5^**:** a measure of centrality that considers the connection patterns among each author’s immediate neighbors. Its value for each author is the probability that a person starting at any random author and randomly selecting links to other authors will arrive at the author in question.

**Layout:** a visual representation of the vertices and edges of a network. Many different layouts can correspond to the same underlying network, and the arrangement of the vertices and edges of the network is usually chosen to be understandable and reflect as closely as possible the true network organization.

**Distributed recursive graph (DrL**^6^**) algorithm:** an algorithm that creates a layout in which vertices with strongly weighted edges are placed close together and weakly weighted edges are pressed apart. The algorithm represents edges as springs, with attractive or repulsive “forces” acting on them relative to the weight of that edge, and seeks to minimize the overall “energy” of the system with its final layout. The distributed recursive graph algorithm (DrL) is intended for very large networks and aims to generate enough whitespace within the layout for the resulting clustering trends to be visible and interpretable.

**Preferential attachment:** the idea that the more connected a vertex already is, the more likely it is to receive new connections. In the context of co-authorship, this is the idea that authors with many co-authorship links are more likely to form new co-authorship links, or to strengthen their existing links, than authors with few existing links.

**Scale-free phenomena:** a network exhibits the scale-free phenomenon when the distribution of its vertex degrees follows a power law, reflective of preferential attachment.

**Q factor**^7^**:** a metric developed to quantify the ability of a scientist to “take advantage of the available knowledge in a way that enhances (*Q* > 1) or diminishes (*Q* < 1) the potential impact *p* of a paper” where the potential impact *p* is random (reflective of an element of luck) and the overall impact of a publication is then *Qp*.

# **Supplemental Methods**

## **Author impact score calculation – examples**

The following examples illustrate the calculation of the author impact score for a variety of scenarios, using the four coefficients of author role, trial type, citations score, and updates:

*John Smith is the first author of the primary report of a phase III RCT, which was published in 1975 and has been cited 1000 times. His impact score for this manuscript is 3 x 2 x 1000/13,341 x 1 = 0.4497.*

*Jane Doe is the middle author of the primary report of a non-randomized trial which was published in 2005 and has been cited 50 times. Her impact score for this manuscript is 1 x 1 x 50/13,341 x 1 = 0.0037.*

*Mary Smith is the last author of the first update of a phase III RCT which was published in an upper tier journal in 2015 and has been cited 50 times. Her impact score for this manuscript is 3 x 2 x (313.6 + 50)/13,341 x 0.5 = 0.0818.*

*George Doe is the middle author of the second update of a non-randomized trial which was published in a lower tier journal in 2018 and has been cited 1 times. His impact score for this manuscript is 1 x 1 x (1 + 79)/13,341 x 0.25 = 0.0015.*

## **Co-authorship score for edge weighting – examples**

The following examples only show the effect of the author role coefficient, for simplicity.

**Example #1:** A manuscript with three authors (one first, one middle, one last). There is one senior-senior co-author relationship, with weight (3 x 3)/3 = 3. There are two middle-senior co-author relationships, with weight (3 x 1)/3 = 1. Total edge weight for this manuscript is therefore 3 + (2 x 1) = 5.

**Example #2:** A manuscript with six authors (one first, four middle, one last). There is one senior-senior co-author relationship, with weight (3 x 3)/6 = 1.5. There are eight middle-senior co-author relationships, with weight (3 x 1)/6 = 0.5. There are six middle-middle co-author relationships, with weight (1 x 1)/6 = $\frac{1}{6}$. Total edge weight for this manuscript is therefore 1.5 + (8 x 0.5) + (6 x $\frac{1}{6}$) = 6.5.

# **Supplemental Figures**


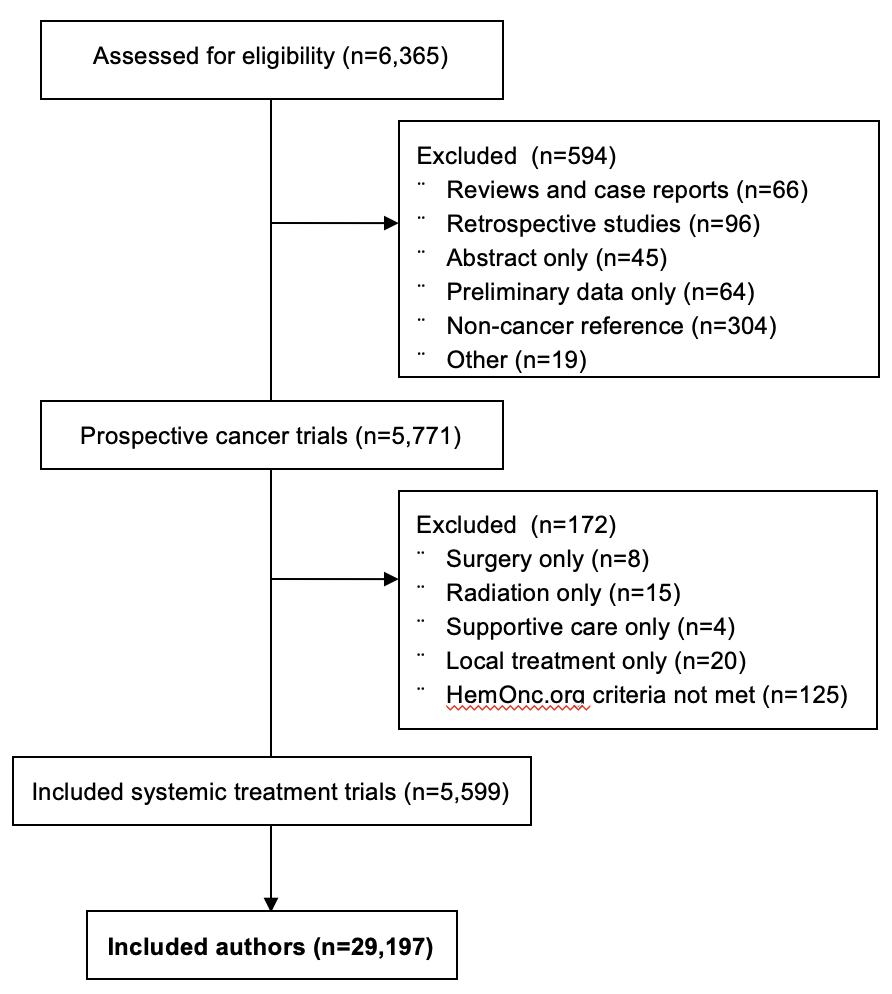


## **Figure S1. CONSORT diagram of author eligibility selection process**

Note that any publication e-published on or after January 1, 2019 was not assessed for eligibility and is not included here. Publications e-published on or prior to December 31, 2018 were eligible, regardless of when the print manuscript was published.


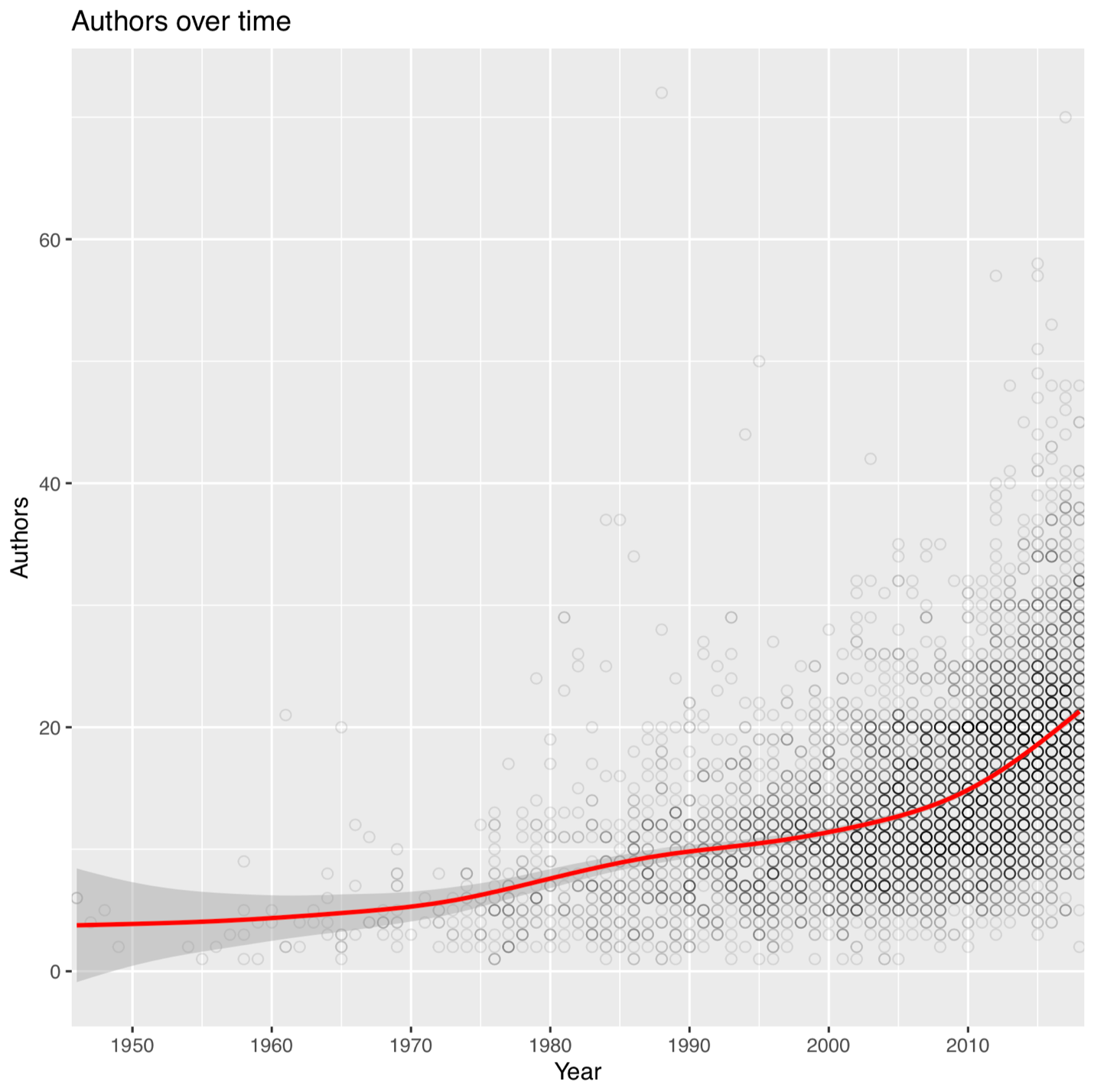


## **Figure S2. Number of authors per publication over time**

The LOESS curve shows a near-linear rise is authors per publication from 1970-2005, followed by a steeper rise over the past 15 years.


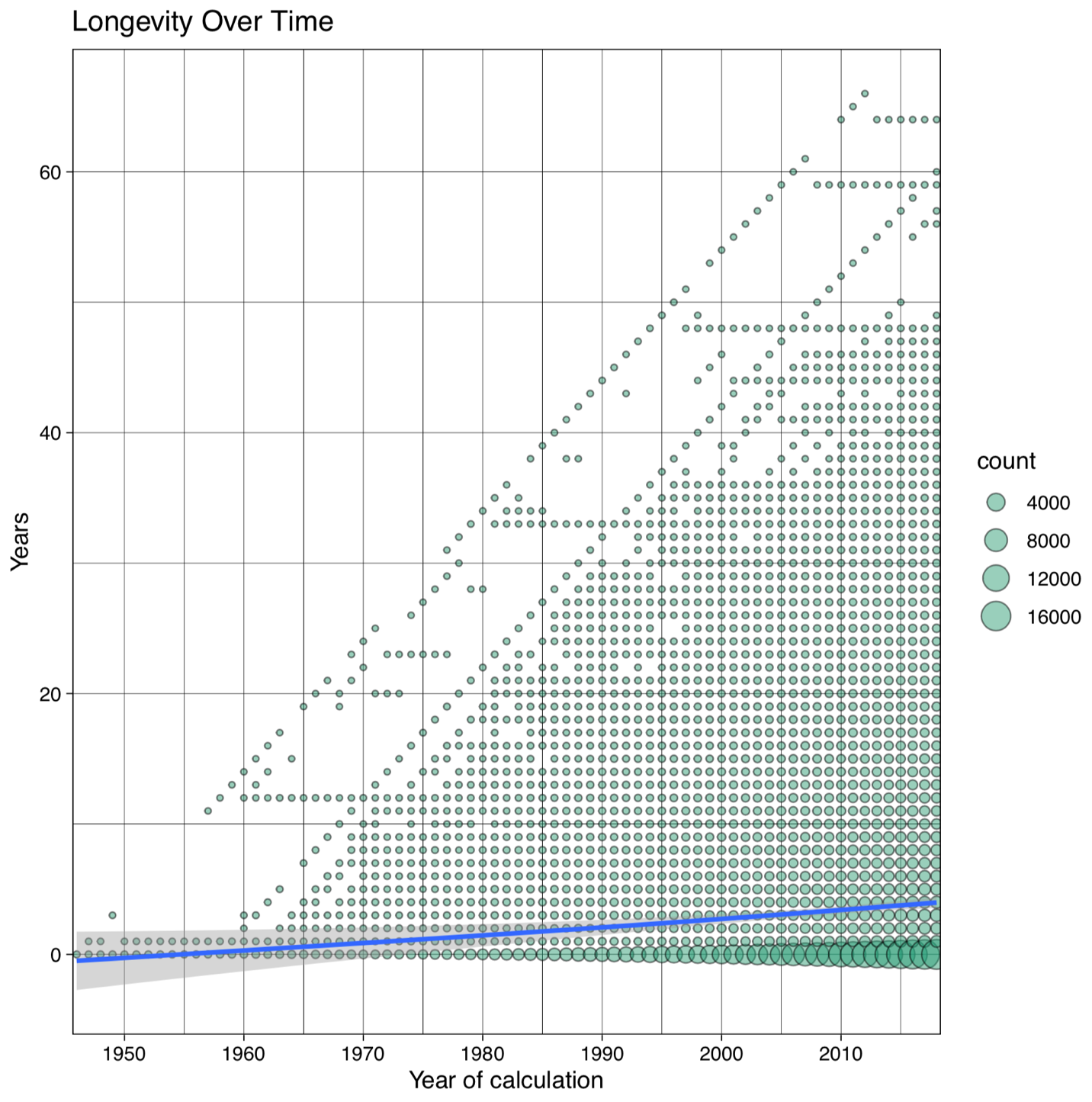


## **Figure S3. Author longevity over time**

The LOESS curve shows a slow but sustained increase in authors with extended longevity, even though the median longevity remains at <1 year across all cumulative years. Horizontal lines (e.g., that at 59 years from 2008 onwards) are not artifacts – these represent authors whose productive period has ended (e.g., due to retirement). Given that the networks are cumulative, these authors will remain at their final longevity.

## **Figure S4. Dominant authors’ share of the field over time**

Given the log-linear growth of the network, the dominant authors’ share of the total impact decreases over time, although the ratio of their impact to the median impact increases.


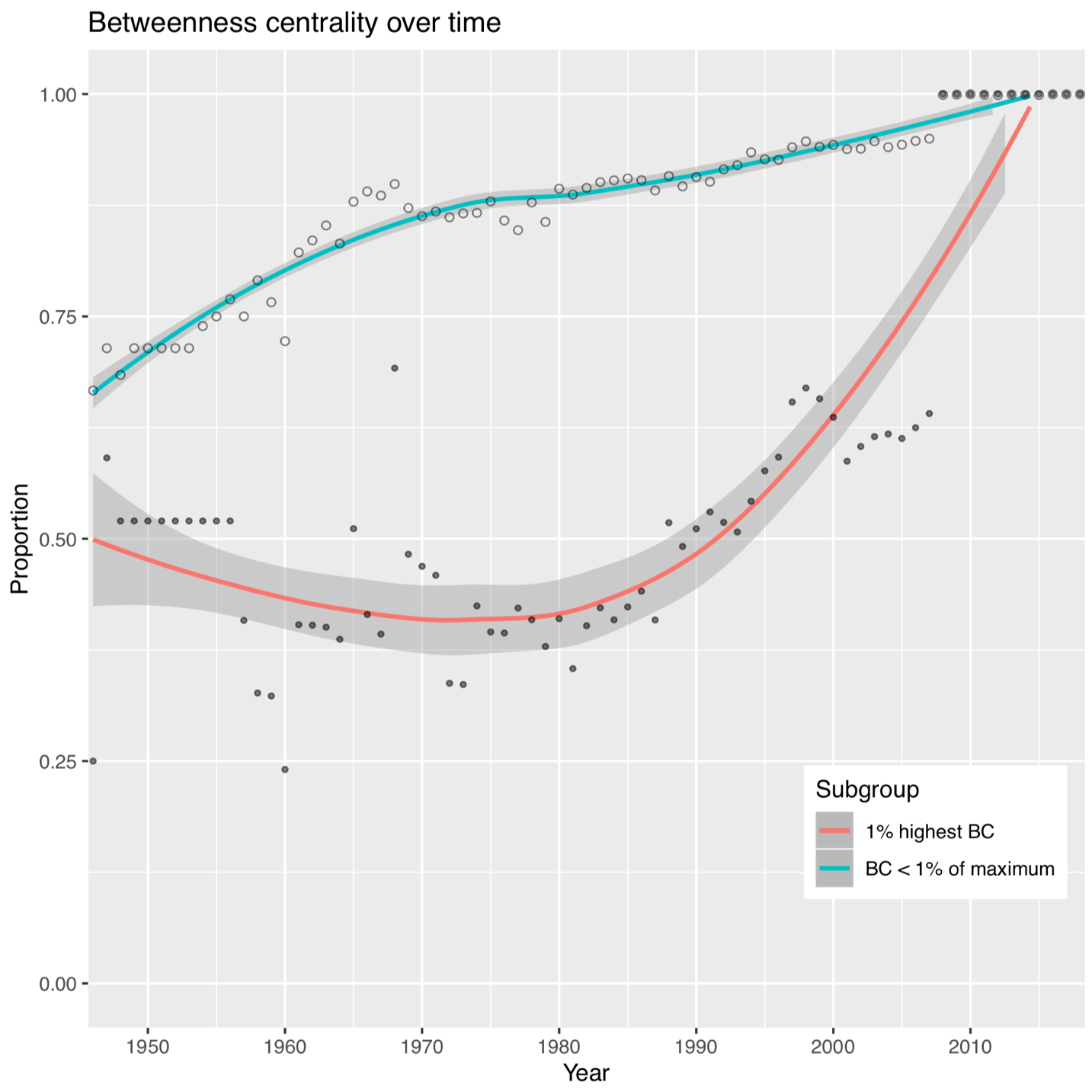


## **Figure S5. Betweenness centrality (BC) over time**

Note that while the y-scale is the same for both curves, they should be interpreted separately. For those with the 1% highest BC, the LOESS curve shows what proportion of total summative BC they have in the network; this proportion has been near 100% for the past 15 years. For those with BC <1% of the maximum, the LOESS curve shows what proportion of authors falls into this category. The extreme disparity in BC is suggestive of scale-free effects.


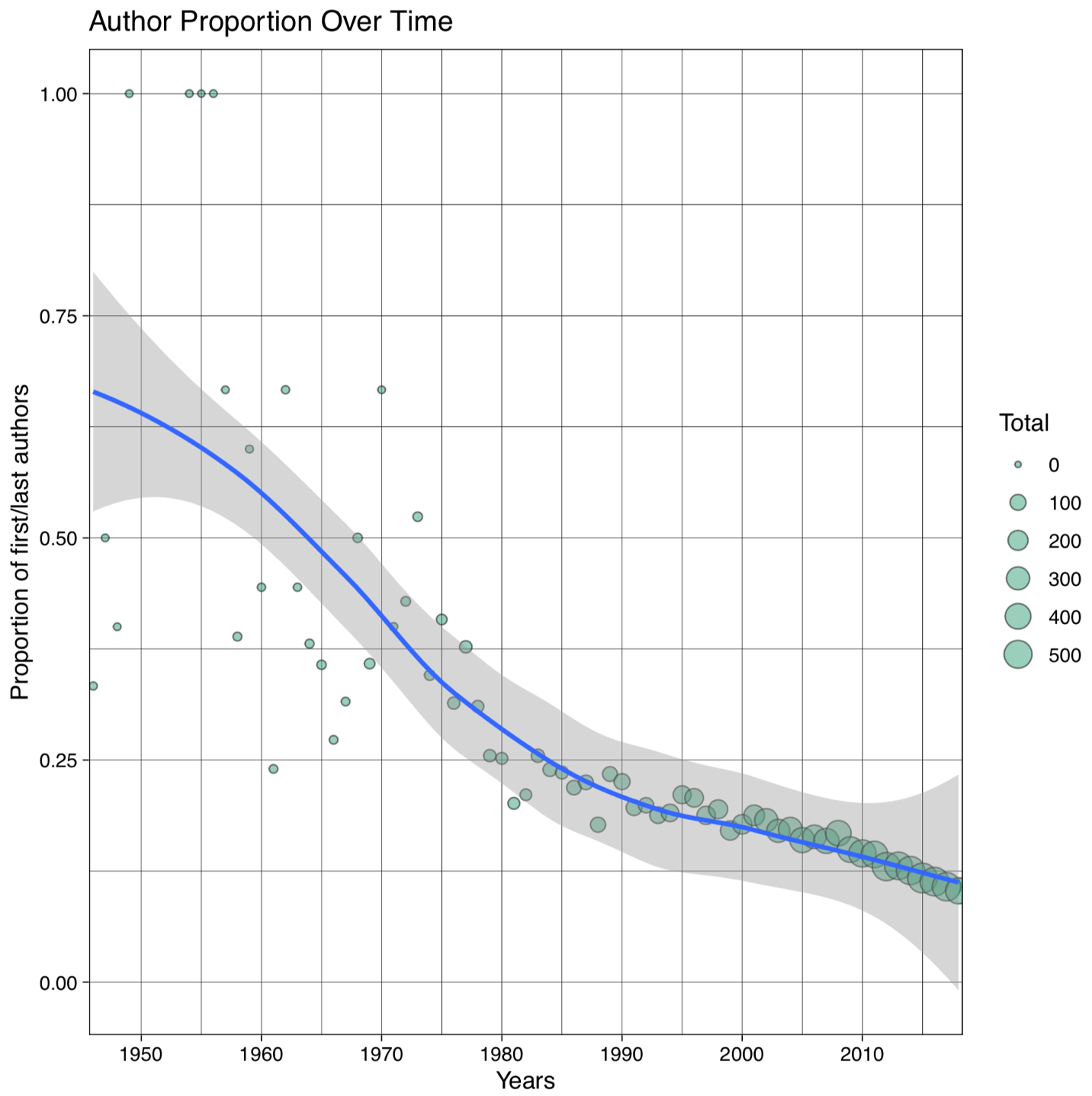


## **Figure S6. Proportion of first/last authorship over time**

The LOESS curve shows a continuous downward trend in the proportion of first/last authorship over time. Note that in contrast to most of the other figures, this one is not showing cumulative data; each year represents manuscripts published in that year.


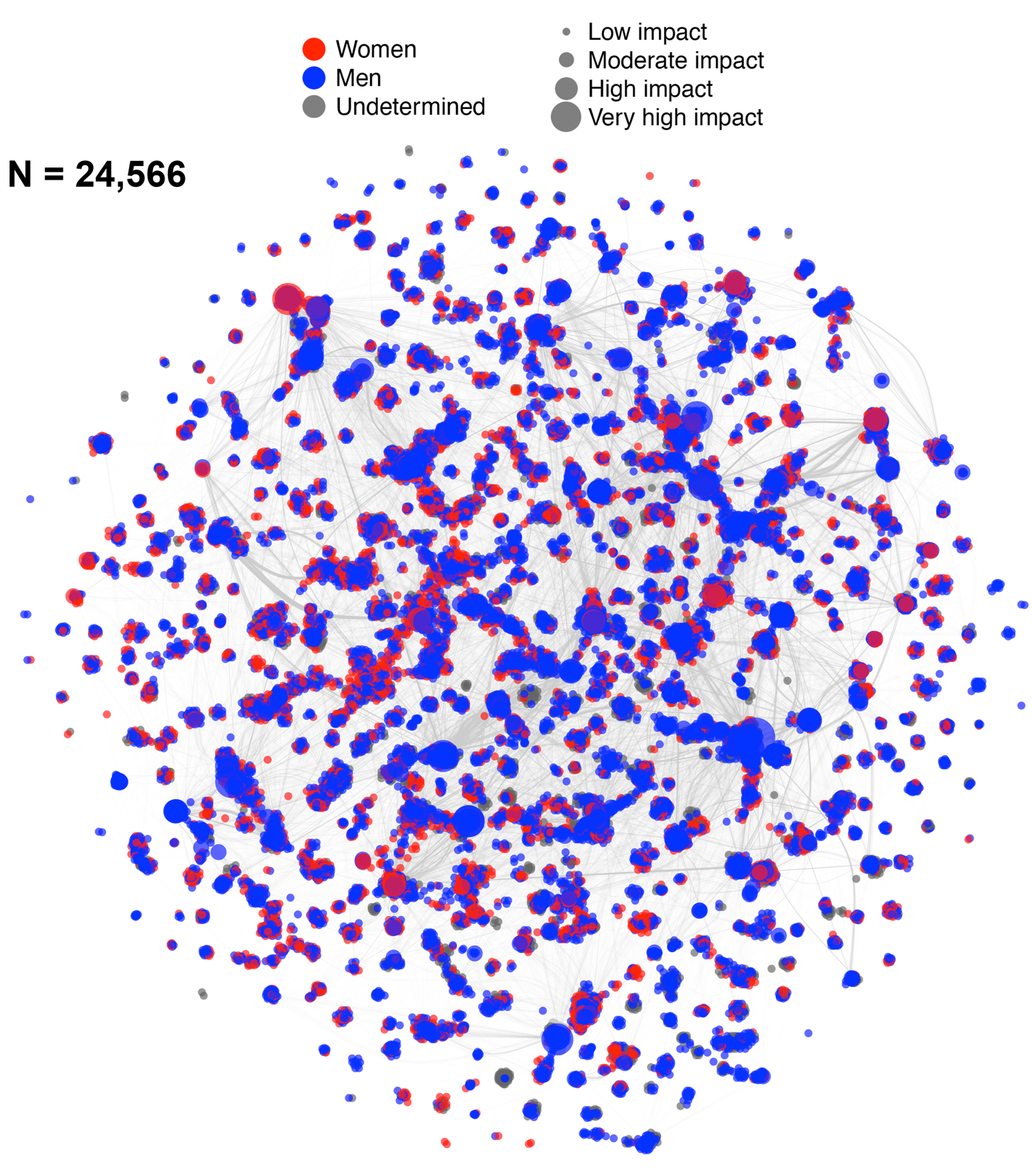


## **Figure S7. Final network visualization, colored by gender.**

## **Figure S8. Density distribution of author impact, 2018**

The author impact scale is logarithmic. Vertical white lines represent the mean ± 1 standard deviation.


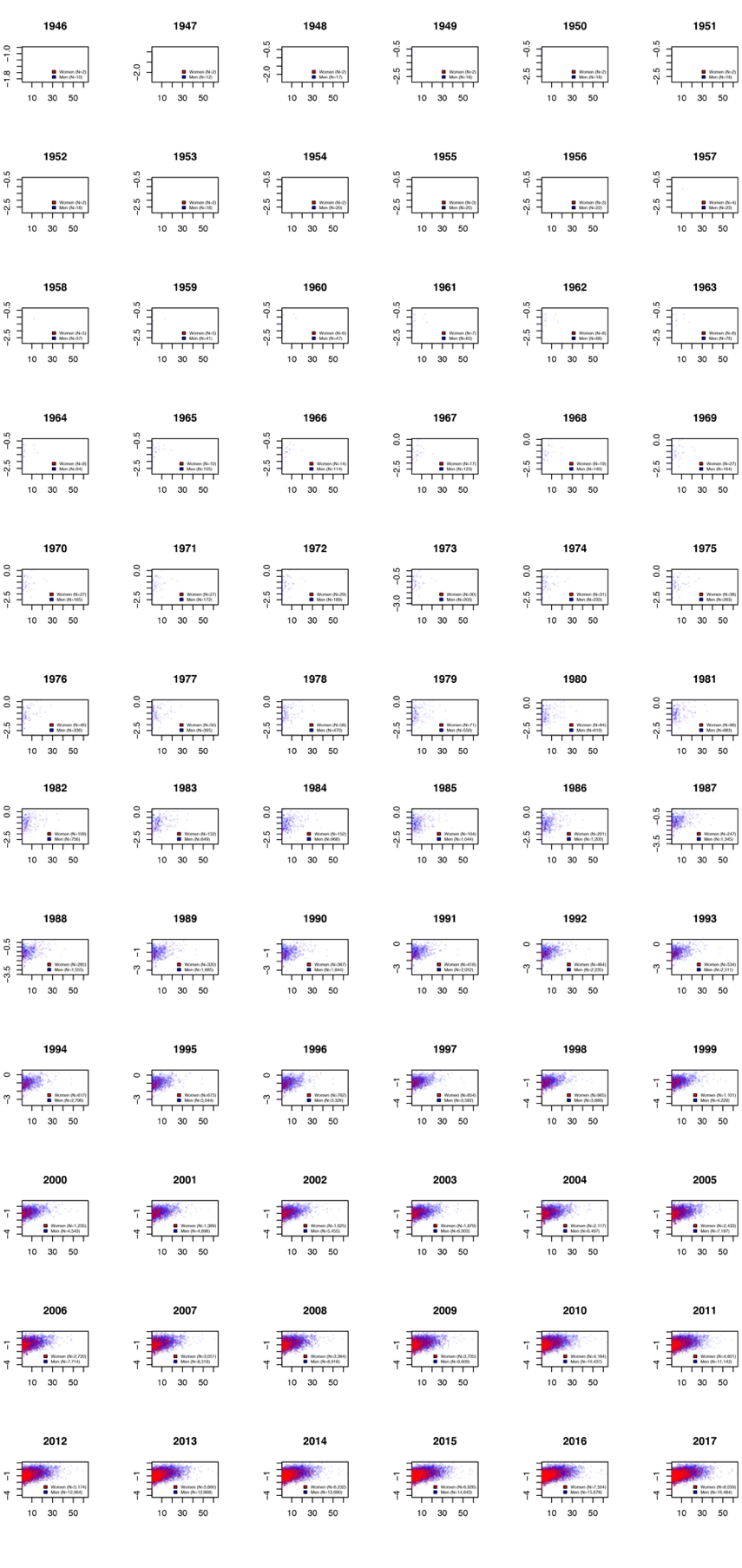


## **Figure S9. Total impact versus longevity, 1946-2017**


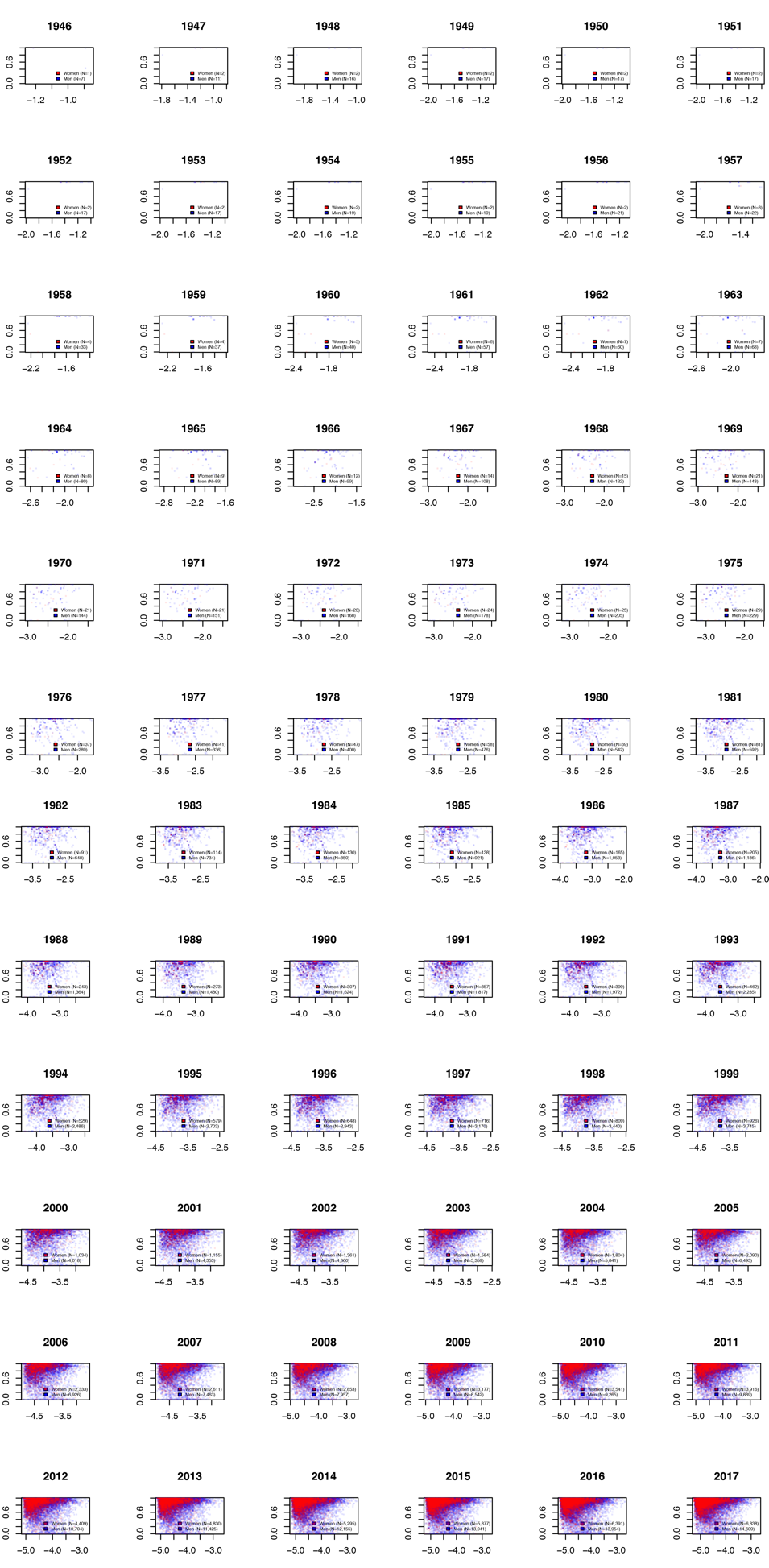


## **Figure S10. Homophily versus PageRank, 1946-2017**


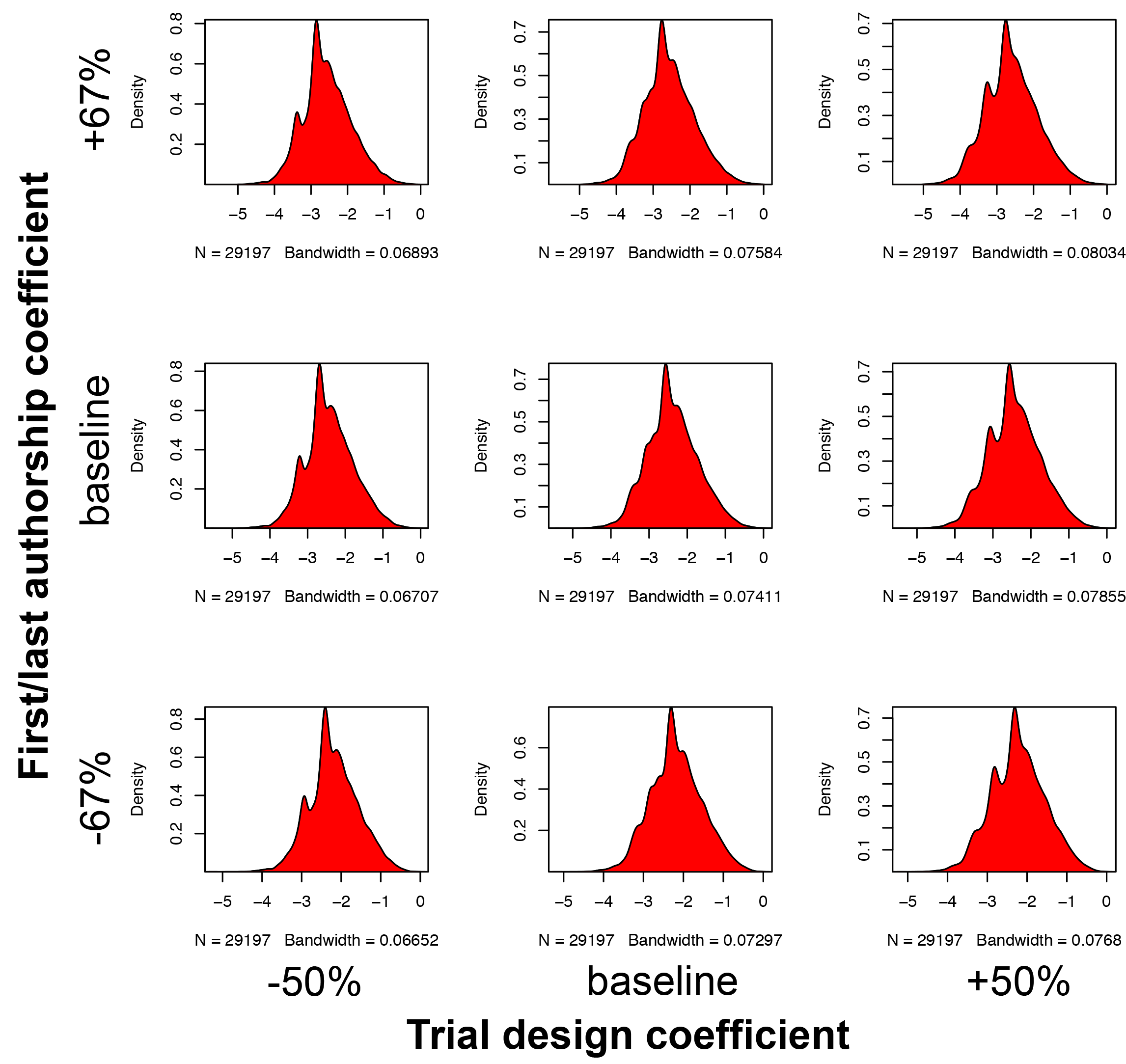


## **Figure S11. Normalized author score distributions, sensitivity analysis**

The permutation shown in each panel is the intersection of the two axes; e.g., the center panel is the baseline analysis, and the bottom left panel is the permutation where the trial design coefficient is reduced by 50% and the author role coefficient is reduced by 67%.


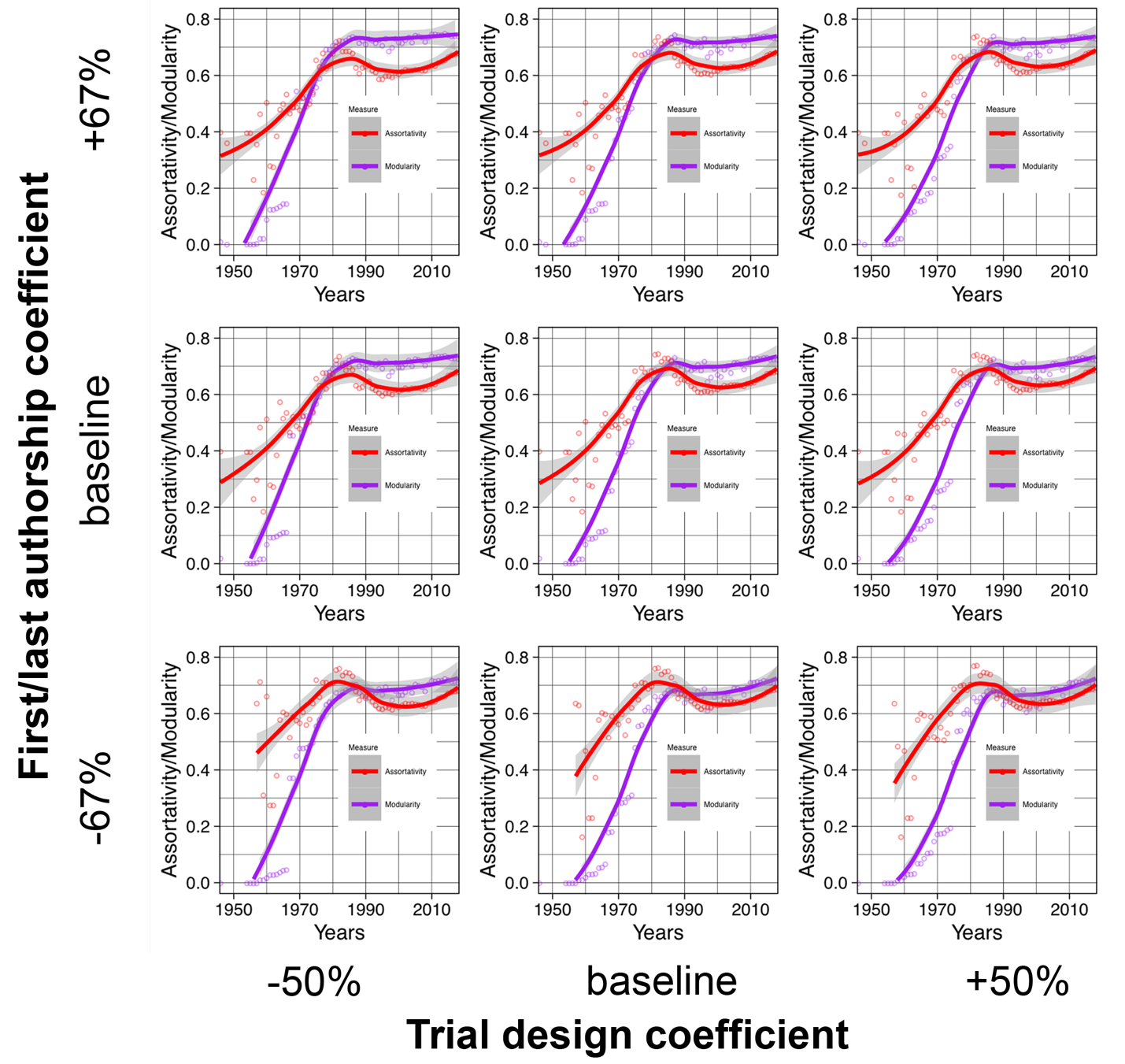


## **Figure S12. Assortativity and modularity, sensitivity analysis**

The permutation shown in each panel is the intersection of the two axes; e.g., the center panel is the baseline analysis, and the bottom left panel is the permutation where the trial design coefficient is reduced by 50% and the author role coefficient is reduced by 67%.


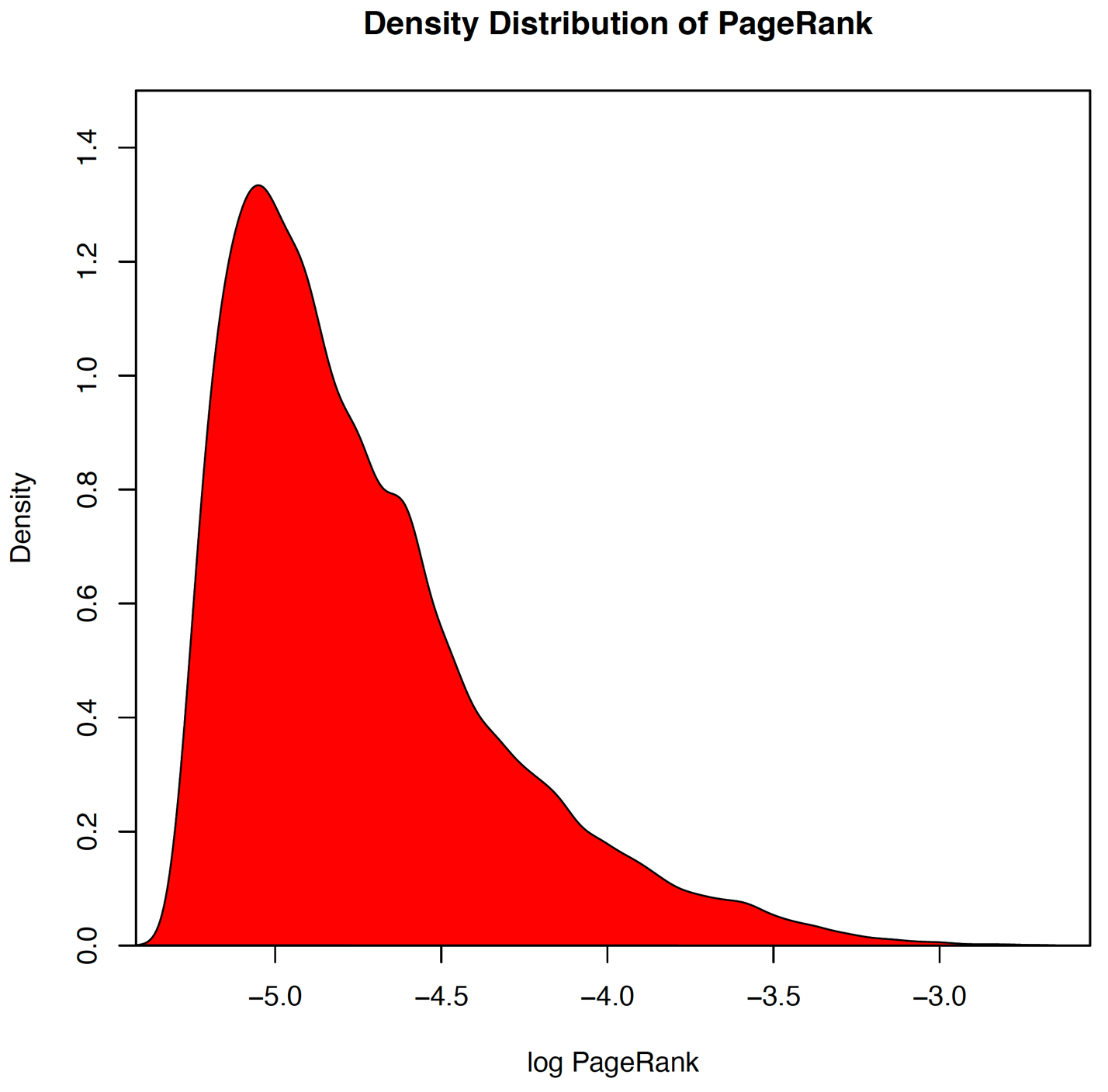


## **Figure S13. Density distribution of PageRank scores, 2018**


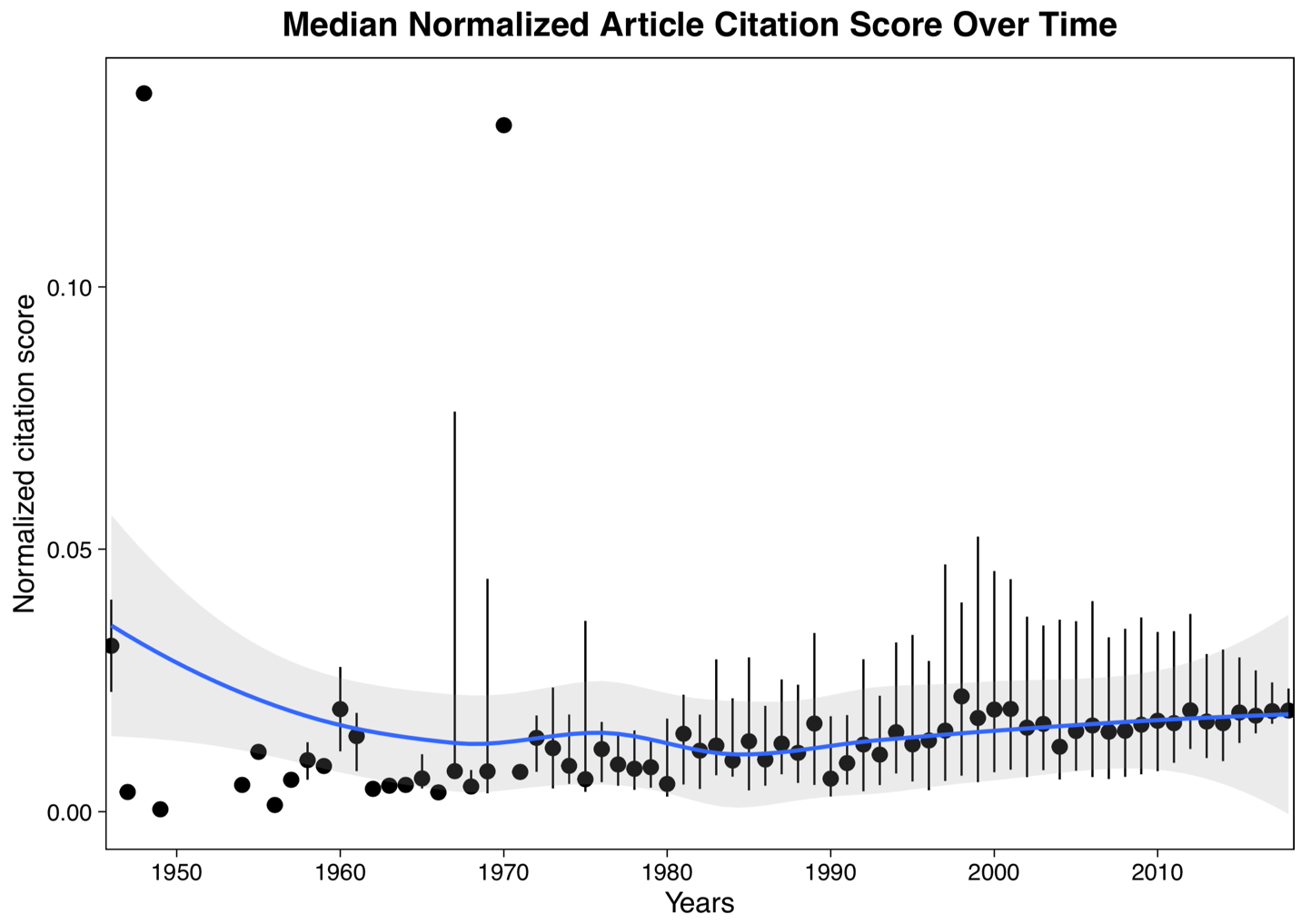


**Figure S14. Article citation score over time**

Black points represent the median and black bars represent the interquartile range for the citation score of articles published in that year; the LOESS curve shows that since 1980 the citation score has been steady-to-slightly increasing. Note that as described in the eMethods, citation score is equivalent to citations for the years 1946-2008, and is a blended score based on citations and journal tier for the years 2009-2018 (see **Table S5**).

# **Supplemental Tables**

## **Table S1. Mapping of HemOnc.org cancer subtype and site-agnostic pages to subspecialties**

| Disease-specific or site-agnostic page MAPS TO: | Subspecialty |
| --- | --- |
| Breast cancer | Breast oncology |
| Breast cancer, ER/PR-positive |  |
| Breast cancer, HER2-positive |  |
| Breast cancer, triple negative |  |
| Breast cancer, BRCA-mutated |  |
| Breast cancer, PIK3CA-mutated |  |
| Adenocarcinoma of unknown primary^a^ |  |
| *BRAF*^b^ |  |
| *EGFR*^b^ |  |
| *ERBB2* (*HER2*)^b^ |  |
| *ERBB3* (*HER3*)^b^ |  |
| *SMO* or *PTCH1* (Hedgehog)^b^ |  |
| Central nervous system melanoma | Dermatologic oncology |
| Cutaneous basal cell carcinoma |  |
| Cutaneous squamous cell carcinoma |  |
| Melanoma |  |
| Melanoma, BRAF-mutated |  |
| Melanoma, NRAS-mutated |  |
| Merkel cell carcinoma |  |
| Uveal melanoma |  |
| Adrenocortical carcinoma | Endocrine oncology |
| Neuroendocrine tumor |  |
| Pancreatic neuroendocrine tumor |  |
| Pheochromocytoma |  |
| Thyroid cancer |  |
| Thyroid cancer, BRAF-mutated |  |
| *BRAF^b^* |  |
| *NTRK^b^* |  |
| Anal cancer | Gastrointestinal oncology |
| Cholangiocarcinoma |  |
| Colon cancer |  |
| Colon cancer, KRAS wild-type |  |
| Esophageal cancer |  |
| Gallbladder cancer |  |
| Gastric cancer | Gastrointestinal oncology, cont. |
| Hepatoblastoma |  |
| Hepatocellular carcinoma |  |
| Pancreatic cancer |  |
| Periampullary adenocarcinoma |  |
| Rectal cancer |  |
| Adenocarcinoma of unknown primary^a^ |  |
| *BRAF^b^* |  |
| *EGFR^b^* |  |
| *ERBB2* (*HER2*)^b^ |  |
| *ERBB3* (*HER3*)^b^ |  |
| MSI-H or dMMR^b^ |  |
| *NTRK^b^* |  |
| *SMO* or *PTCH1* (Hedgehog)^b^ |  |
| Bladder cancer | Genitourinary oncology |
| Neuroblastoma |  |
| Penile cancer |  |
| Prostate cancer |  |
| Renal cell carcinoma |  |
| Testicular cancer |  |
| Wilms tumor |  |
| Adenocarcinoma of unknown primary^a^ |  |
| *BRAF^b^* |  |
| *EGFR^b^* |  |
| *ERBB2* (*HER2*)^b^ |  |
| *ERBB3* (*HER3*)^b^ |  |
| *SMO* or *PTCH1* (Hedgehog)^b^ |  |
| Cervical cancer | Gynecologic oncology |
| Endometrial cancer |  |
| Gestational trophoblastic neoplasia |  |
| Ovarian cancer |  |
| Vulvar cancer |  |
| Adenocarcinoma of unknown primary^a^ |  |
| *BRAF^b^* |  |
| *EGFR^b^* |  |
| *ERBB2* (*HER2*)^b^ |  |
| *ERBB3* (*HER3*)^b^ |  |
| MSI-H or dMMR^b^ |  |
| *SMO* or *PTCH1* (Hedgehog)^b^ | Gynecologic oncology, cont. |
| Head and neck cancer | Head and neck oncology |
| Nasopharyngeal carcinoma |  |
| Oropharyngeal cancer |  |
| *BRAF^b^* |  |
| *EGFR^b^* |  |
| *ERBB2* (*HER2*)^b^ |  |
| *NTRK^b^* |  |
| *SMO* or *PTCH1* (Hedgehog)^b^ |  |
| Adult T-cell leukemia-lymphoma | Lymphoid malignancy |
| Anaplastic large cell lymphoma |  |
| B-cell acute lymphoblastic leukemia |  |
| B-cell acute lymphoblastic leukemia, Ph+ |  |
| Burkitt lymphoma |  |
| Chronic lymphocytic leukemia |  |
| Central nervous system lymphoma |  |
| Classic Hodgkin lymphoma |  |
| Cutaneous T-cell lymphoma |  |
| Diffuse large B-cell lymphoma |  |
| Extranodal NK-T-cell lymphoma nasal type |  |
| Follicular lymphoma |  |
| Hairy cell leukemia |  |
| HIV-associated lymphoma |  |
| Hodgkin lymphoma nodular lymphocyte-predominant |  |
| Mantle cell lymphoma |  |
| Marginal zone lymphoma |  |
| Mediastinal gray-zone lymphoma |  |
| NK-T-cell lymphoma |  |
| Peripheral T-cell lymphoma |  |
| Post-transplant lymphoproliferative disorder |  |
| Primary mediastinal B-cell lymphoma |  |
| T-cell acute lymphoblastic leukemia |  |
| Transformed lymphoma |  |
| Allogeneic stem cell transplant^a^ |  |
| Acute myeloid leukemia | Myeloid malignancy |
| Acute myeloid leukemia, FLT3-positive |  |
| Acute myeloid leukemia, IDH-mutated |  |
| Acute promyelocytic leukemia |  |
| Aplastic anemia | Myeloid malignancy, cont. |
| Blastic plasmacytoid dendritic cell neoplasm |  |
| Chronic myelogenous leukemia |  |
| Chronic myelomonocytic leukemia |  |
| Central nervous system leukemia |  |
| Erdheim-Chester disease |  |
| Essential thrombocythemia |  |
| Hypereosinophilic syndrome |  |
| Langerhans cell histiocytosis |  |
| Myelodysplastic syndrome |  |
| Myelofibrosis |  |
| Polycythemia vera |  |
| Rosai-Dorfman-Destombes disease |  |
| Systemic mastocytosis |  |
| Allogeneic stem cell transplant^a^ |  |
| *BRAF^b^* |  |
| Anaplastic glioma | Neuro-oncology |
| CNS carcinoma |  |
| Glioblastoma |  |
| Low-grade glioma |  |
| Medulloblastoma |  |
| Meningioma |  |
| Light-chain (AL) amyloidosis | Plasma cell disorders |
| Multiple myeloma |  |
| Plasma cell leukemia |  |
| POEMS syndrome |  |
| Waldenström macroglobulinemia |  |
| *BRAF^b^* |  |
| Bone sarcoma | Sarcoma |
| Ewing sarcoma |  |
| Gastrointestinal stromal tumor |  |
| Osteosarcoma |  |
| Rhabdomyosarcoma |  |
| Soft tissue sarcoma |  |
| Vascular sarcoma |  |
| *NTRK^b^* |  |
| Mesothelioma | Thoracic oncology |
| Non-small cell lung cancer |  |
| Non-small cell lung cancer, ALK-positive | Thoracic oncology, cont. |
| Non-small cell lung cancer, BRAF-mutated |  |
| Non-small cell lung cancer, EGFR-mutated |  |
| Non-small cell lung cancer, ROS1-positive |  |
| Small cell lung cancer |  |
| Thymoma |  |
| Adenocarcinoma of unknown primary^a^ |  |
| *BRAF^b^* |  |
| *EGFR^b^* |  |
| *ERBB2* (*HER2*)^b^ |  |
| *ERBB3* (*HER3*)^b^ |  |
| *SMO* or *PTCH1* (Hedgehog)^b^ |  |
| Acquired coagulopathy | Unmapped^c^ |
| Antiphospholipid antibody syndrome |  |
| Atypical hemolytic uremic syndrome |  |
| Autoimmune cytopenia |  |
| Autologous stem cell transplant |  |
| Castleman disease |  |
| Cold agglutinin disease |  |
| Graft versus host disease |  |
| Hemophagocytic lymphohistiocytosis |  |
| Heparin-induced thrombocytopenia |  |
| Hereditary hemorrhagic telangiectasia |  |
| Immune thrombocytopenia |  |
| Inherited coagulopathy |  |
| Large granular lymphocytic leukemia |  |
| Paroxysmal nocturnal hemoglobinuria |  |
| Sickle cell anemia |  |
| Stem cell mobilization |  |
| Thrombocytopenia in liver disease |  |
| Thrombotic thrombocytopenic purpura |  |
| Venous thromboembolism |  |

^a^These disease-agnostic pages were mapped at the individual reference level; see **Table S6**.

^b^These pages are organized by molecular target and were mapped at the individual reference level; see **Table S6**.

^c^Unmapped pages were removed from the analysis.

BRAF: B-Raf proto-oncogene, serine/threonine kinase; dMMR: deficient mismatch repair; EGFR: epidermal growth factor receptor; ERBB2/HER2: erb-b2 receptor tyrosine kinase 2; ERBB3/HER3: erb-b2 receptor tyrosine kinase 3; MSI-H: microsatellite instability-high; NTRK: neurotrophic receptor tyrosine kinase; POEMS: polyneuropathy, organomegaly, endocrinopathy, monoclonal gammopathy, skin changes; PTCH1: patched 1; SMO: smoothened, frizzled class receptor

## **Table S2. Study characteristics for the final cumulative network**

| Characteristics | Publications, No. (%)^a^ | Authors per publication, median (IQR) | Authors, No. (%)^a^ | Gender %, man/woman/unknown |
| --- | --- | --- | --- | --- |
| **Trial design** | | | | |

| Randomized clinical trial (RCT) | 3,840 (68.6) | 18 (13-23) | 22,761 (78) | 61/27/11 |
| --- | --- | --- | --- | --- |
| “Positive” RCT^b^ | 2,131 (55.5) | 19 (14-24) | 15,340 (52.5) | 65/28/7 |
| “Negative” RCT^c^ | 1,534 (39.9) | 17 (12-21) | 12,474 (42.7) | 65/25/10 |
| Unknown outcome RCT^d^ | 175 (4.6) | 18 (13-22) | 2,141 (7.3) | 67/26/7 |
| Non-randomized | 1,759 (31.4) | 15 (11-20) | 11,687 (40) | 60/31/8 |

| Publication type |
| --- |

| Primary | 4,800 (85.7) | 17 (12-22) | 28,087 (96.2) | 60/29/12 |
| --- | --- | --- | --- | --- |
| Update | 799 (14.3) | 18 (13-22) | 6,773 (23.2) | 67/30/3 |

| Journal |
| --- |

| *Journal of Clinical Oncology* | 1,595 (28.5) | 13 (10-18) | 12,140 (41.6) | 66/28/6 |
| --- | --- | --- | --- | --- |
| *Lancet* journals^e^ | 710 (12.7) | 19 (13-22) | 8,193 (28.1) | 68/30/1 |
| *New England Journal of Medicine* | 495 (8.8) | 15 (10-23) | 5,665 (19.4) | 70/29/0 |
| *Blood* journals^f^ | 495 (8.8) | 16 (11-20) | 4,167 (14.3) | 63/34/3 |
| Other | 2,304 (41.2) | 11 (8-16) | 16,458 (56.4) | 59/25/16 |

| Subspecialty |
| --- |

| Lymphoid malignancy | 1,229 (21.5) | 13 (9-19) | 7,134 (24.4) | 60/32/8 |
| --- | --- | --- | --- | --- |

| Breast oncology | 810 (14.2) | 12.5 (9-18) | 4,756 (16.3) | 57/31/12 |
| --- | --- | --- | --- | --- |

| Gastrointestinal oncology | 757 (13.2) | 14 (10-19) | 5,736 (19.6) | 64/22/14 |
| --- | --- | --- | --- | --- |
| Thoracic oncology | 598 (10.5) | 14 (10-18) | 3,886 (13.3) | 65/24/11 |
| Myeloid malignancy | 520 (9.1) | 15 (11-20) | 3,603 (12.3) | 63/30/7 |
| Genitourinary oncology | 467 (8.2) | 12 (8-17) | 2,997 (10.3) | 69/24/8 |
| Plasma cell disorders | 399 (7) | 16 (10-21) | 2,527 (8.7) | 59/34/6 |
| Gynecologic oncology | 302 (5.3) | 10 (7-15) | 1,870 (6.4) | 60/29/11 |
| Dermatologic oncology | 174 (3) | 16 (11-22) | 1,258 (4.3) | 64/30/6 |
| Sarcoma | 144 (2.5) | 13 (10-19) | 1,163 (4) | 64/30/6 |

| Head and neck oncology | 136 (2.4) | 13 (10-19) | 1,360 (4.7) | 67/24/9 |
| --- | --- | --- | --- | --- |

| Neuro-oncology | 116 (2) | 13.5 (11-21) | 1,097 (3.8) | 68/28/4 |
| --- | --- | --- | --- | --- |
| Endocrine oncology | 66 (1.2) | 13 (7.25-19) | 621 (2.1) | 63/30/7 |

| Total | 5,599 (100) | 13 (9-19) | 29,197 (100) | 59/29/12 |
| --- | --- | --- | --- | --- |

^a^Percentages may not total 100 because of rounding or because a publication and/or author can belong to more than one category.

^b^RCTs where there is a statistically significant primary outcome, defined as p-value ≤0.10 for a superiority design or meeting the prespecified boundary for a non-inferiority or equivalence design.

^c^RCTs with a control arm where there is not a statistically signifcant primary outcome, defined as p-value >0.10 for a superiority design or failing to meet the prespecified boundary for a non-inferiority or equivalence design.

^d^RCTs with no outcome reported or not yet curated on HemOnc.org.

^e^Includes *Lancet*, *Lancet Diabetes & Endocrinology*, *Lancet Gastroenterology & Hepatology*, *Lancet Haematology*, *Lancet Oncology*, and *Lancet Respiratory Medicine*

^f^Includes *Blood* and *Blood Advances*

## **Table S3. Sensitivity analysis results**

| **Author role coefficient (adjustment)** | **Trial design coefficient** | **Assortativity, *r*** | **Modularity, *r*** |
| --- | --- | --- | --- |
| 1 or 5 (+67%) | 1 (-50%) | 0.975 | 0.987 |
| 1 or 5 (+67%) | 1 or 2 (baseline) | 0.977 | 0.999 |
| 1 or 5 (+67%) | 1 or 3 (+50%) | 0.971 | 0.995 |
| 1 or 3 (baseline) | 1 (-50%) | 0.996 | 0.988 |
| 1 or 3 (baseline) | 1 or 2 (baseline) | 1.000 | 1.000 |
| 1 or 3 (baseline) | 1 or 3 (+50%) | 0.999 | 0.991 |
| 1 (-67%) | 1 (-50%) | 0.815 | 0.991 |
| 1 (-67%) | 1 or 2 (baseline) | 0.824 | 0.995 |
| 1 (-67%) | 1 or 3 (+50%) | 0.827 | 0.981 |

## **Table S4. Journal tiers of the 195 journals with publications in the analyzed database**

| Journal title^a^ | Newer title^b^ | Tier^c^ |
| --- | --- | --- |
| J Am Med Assoc | JAMA | Upper |
| JAMA |  |  |
| Lancet |  |  |
| N Engl J Med |  |  |
| Nature |  |  |
| Science |  |  |
| Ann Intern Med |  | Middle |
| Ann Neurol |  |  |
| Ann Oncol |  |  |
| Arch Intern Med |  |  |
| Blood |  |  |
| Br Med J |  |  |
| Cancer Discov |  |  |
| Clin Cancer Res |  |  |
| Eur Urol |  |  |
| Gut |  |  |
| Hepatology |  |  |
| J Allergy Clin Immunol |  |  |
| J Clin Oncol |  |  |
| J Hepatol |  |  |
| J Natl Cancer Inst |  |  |
| J Natl Cancer Inst Monogr | J Natl Cancer Inst |  |
| J Thorac Oncol |  |  |
| JAMA Oncol |  |  |
| Lancet Diabetes Endocrinol |  |  |
| Lancet Gastroenterol Hepatol |  |  |
| Lancet Haematol |  |  |
| Lancet Oncol |  |  |
| Lancet Respir Med |  |  |
| Leukemia |  |  |
| Nat Med |  |  |
| Sci Transl Med |  |  |
| Acta Oncol |  | Lower |
| Adv Ther |  |  |
| AJR Am J Roentgenol |  |  |
| Am J Clin Oncol |  |  |
| Am J Hematol |  | Lower, cont. |
| Am J Med |  |  |
| Am J Obstet Gynecol |  |  |
| Am J Pediatr Hematol Oncol |  |  |
| Am J Roentgenol Radium Ther Nucl Med | AJR Am J Roentgenol |  |
| Am J Transplant |  |  |
| Ann Hematol |  |  |
| Ann Surg |  |  |
| Ann Surg Oncol |  |  |
| Ann Transl Med |  |  |
| Anticancer Drugs |  |  |
| Anticancer Res |  |  |
| Arch Dermatol |  |  |
| Asia Pac J Clin Oncol |  |  |
| Asian J Urol |  |  |
| Asian Pac J Cancer Prev |  |  |
| Biol Blood Marrow Transplant |  |  |
| Biomed Pharmacother |  |  |
| BJU Int |  |  |
| Blood Adv |  |  |
| Blood Cancer J |  |  |
| BMC Cancer |  |  |
| BMC Med |  |  |
| Bone Marrow Transplant |  |  |
| Br J Cancer |  |  |
| Br J Dermatol |  |  |
| Br J Haematol |  |  |
| Br J Obstet Gynaecol |  |  |
| Br J Surg |  |  |
| Br J Urol | BJU Int |  |
| Breast Cancer |  |  |
| Breast Cancer Res |  |  |
| Breast Cancer Res Treat |  |  |
| Bull Cancer |  |  |
| Can Med Assoc J |  |  |
| Cancer |  |  |
| Cancer Chemother Pharmacol |  |  |
| Cancer Chemother Rep |  |  |
| Cancer Clin Trials | Am J Clin Oncol | Lower, cont. |
| Cancer Commun (Lond) |  |  |
| Cancer Immunol Immunother |  |  |
| Cancer Invest |  |  |
| Cancer J |  |  |
| Cancer J Sci Am |  |  |
| Cancer Med |  |  |
| Cancer Res |  |  |
| Cancer Res Treat |  |  |
| Cancer Sci |  |  |
| Cancer Treat Rep |  |  |
| Chest |  |  |
| Chin J Cancer |  |  |
| Clin Breast Cancer |  |  |
| Clin Colorectal Cancer |  |  |
| Clin Genitourin Cancer |  |  |
| Clin Infect Dis |  |  |
| Clin Lung Cancer |  |  |
| Clin Lymphoma | Clin Lymphoma Myeloma Leuk |  |
| Clin Lymphoma Myeloma | Clin Lymphoma Myeloma Leuk |  |
| Clin Lymphoma Myeloma Leuk |  |  |
| Clin Ther |  |  |
| Dig Dis Sci |  |  |
| Dis Esophagus |  |  |
| Drug Des Devel Ther |  |  |
| EClinicalMedicine |  |  |
| Endocr Relat Cancer |  |  |
| ESMO Open |  |  |
| Eur J Cancer |  |  |
| Eur J Cancer Clin Oncol | Eur J Cancer |  |
| Eur J Haematol |  |  |
| Eur J Surg Oncol |  |  |
| Exp Hematol |  |  |
| Future Oncol |  |  |
| Gastric Cancer |  |  |
| Gynecol Oncol |  |  |
| Haematol Blood Transfus |  |  |
| Haematologica |  |  |
| Head Neck |  | Lower, cont. |
| Health Qual Life Outcomes |  |  |
| Hematol J | Haematologica |  |
| Hematol Oncol |  |  |
| Hematol Oncol Clin North Am |  |  |
| Hematology |  |  |
| Int J Cancer |  |  |
| Int J Clin Oncol |  |  |
| Int J Colorectal Dis |  |  |
| Int J Gynecol Cancer |  |  |
| Int J Hematol |  |  |
| Int J Immunopharmacol |  |  |
| Int J Oncol |  |  |
| Int J Radiat Oncol Biol Phys |  |  |
| Interact Cardiovasc Thorac Surg |  |  |
| Invest New Drugs |  |  |
| J Am Acad Dermatol |  |  |
| J BUON |  |  |
| J Cancer Res Clin Oncol |  |  |
| J Chemother |  |  |
| J Clin Endocrinol Metab |  |  |
| J Coll Physicians Surg Pak |  |  |
| J Gastroenterol |  |  |
| J Geriatr Oncol |  |  |
| J Hematol Oncol |  |  |
| J Immunother |  |  |
| J Immunother Cancer |  |  |
| J Interferon Cytokine Res |  |  |
| J Mt Sinai Hosp N Y |  |  |
| J Neurooncol |  |  |
| J Neurosurg |  |  |
| J Pediatr |  |  |
| J Surg Oncol |  |  |
| J Thorac Cardiovasc Surg |  |  |
| J Thromb Haemost |  |  |
| J Transl Med |  |  |
| J Urol |  |  |
| Jpn J Clin Oncol |  |  |
| Leuk Lymphoma |  | Lower, cont. |
| Leuk Res |  |  |
| Lung Cancer |  |  |
| Med Oncol |  |  |
| Med Pediatr Oncol | Pediatr Blood Cancer |  |
| Medicine (Baltimore) |  |  |
| Melanoma Res |  |  |
| Mol Ther |  |  |
| Neoplasia |  |  |
| Neth J Med |  |  |
| Neuro Oncol |  |  |
| Neurology |  |  |
| Neurosurg Focus |  |  |
| Obstet Gynecol |  |  |
| Oncologist |  |  |
| Oncology |  |  |
| Oncology (Williston Park) |  |  |
| Oncotarget |  |  |
| Onkologie |  |  |
| Pediatr Blood Cancer |  |  |
| Pediatrics |  |  |
| PLoS One |  |  |
| Proc Natl Acad Sci U S A |  |  |
| Prostate Cancer Prostatic Dis |  |  |
| Qual Life Res |  |  |
| Radiother Oncol |  |  |
| Recenti Prog Med |  |  |
| Respirology |  |  |
| Sarcoma |  |  |
| Scand J Haematol | Eur J Haem |  |
| Scand J Urol Nephrol |  |  |
| Sci Rep |  |  |
| Semin Hematol |  |  |
| Semin Oncol |  |  |
| Strahlenther Onkol |  |  |
| Surg Gynecol Obstet |  |  |
| Surg Today |  |  |
| Surgery |  |  |
| Target Oncol |  | Lower, cont. |
| Tumori |  |  |
| Urol Oncol |  |  |
| Urology |  |  |
| World J Gastroenterol |  |  |
| World J Urol |  |  |
| Zhonghua Xue Ye Xue Za Zhi |  |  |

^a^Journal titles are abbreviated in standard ISO 4 format.

^b^Discontinued titles were mapped to their newer counterpart, when applicable.

^c^Journals in the upper tier had an impact factor (IF) of at least 40 and are high-impact general medical journals; journals in the middle tier had an IF of 10 to 40 and are high-impact subspecialty journals; and journals in the lower tier had an IF of less than 10, as of 2019. If the IF was not available, assignment to tier was by consensus of the co-authors.

## **Table S5. Adjusted citation score for manuscripts published between 2009-2018**

| **Year** | **Phase-in** | **Upper tier^a^** | **Middle tier^a^** | **Lower tier^a^** |
| --- | --- | --- | --- | --- |
| 2009 | 10% | 44.8 | 24 | 7.9 |
| 2010 | 20% | 89.6 | 48 | 15.8 |
| 2011 | 30% | 134.4 | 72 | 23.7 |
| 2012 | 40% | 179.2 | 96 | 31.6 |
| 2013 | 50% | 224 | 120 | 39.5 |
| 2014 | 60% | 268.8 | 144 | 47.4 |
| 2015 | 70% | 313.6 | 168 | 55.3 |
| 2016 | 80% | 358.4 | 192 | 63.2 |
| 2017 | 90% | 403.2 | 216 | 71.1 |
| 2018 | **100%** | **448** | **240** | **79** |

^a^This number is added to the citation count of the manuscript. In 2018, this number represents the median number of citations for all articles in the database published in that journal tier between 1946-2008. See **Table S4** for mapping of journals to tiers.

## **Table S6. Re-mapping of site-agnostic references**

| Reference | Trial type | Re-mapping |
| --- | --- | --- |
| Briasoulis et al. 2000^8^ | Adenocarcinoma of unknown primary | Breast oncology; Gastrointestinal oncology; Genitourinary oncology; Gynecologic oncology; Thoracic oncology |
| GEFCAPI 01^9^ | Adenocarcinoma of unknown primary | Breast oncology; Gastrointestinal oncology; Genitourinary oncology; Gynecologic oncology; Thoracic oncology |
| GEFCAPI 02^10^ | Adenocarcinoma of unknown primary | Breast oncology; Gastrointestinal oncology; Genitourinary oncology; Gynecologic oncology; Thoracic oncology |
| Greco et al. 2000a^11^ | Adenocarcinoma of unknown primary | Breast oncology; Gastrointestinal oncology; Genitourinary oncology; Gynecologic oncology; Thoracic oncology |
| Greco et al. 2000b^12^ | Adenocarcinoma of unknown primary | Breast oncology; Gastrointestinal oncology; Genitourinary oncology; Gynecologic oncology; Thoracic oncology |
| Greco et al. 2002^13^ | Adenocarcinoma of unknown primary | Breast oncology; Gastrointestinal oncology; Genitourinary oncology; Gynecologic oncology; Thoracic oncology |
| Hainsworth et al 1992^14^ | Adenocarcinoma of unknown primary | Breast oncology; Gastrointestinal oncology; Genitourinary oncology; Gynecologic oncology; Thoracic oncology |
| Hainsworth et al. 2007^15^ | Adenocarcinoma of unknown primary | Breast oncology; Gastrointestinal oncology; Genitourinary oncology; Gynecologic oncology; Thoracic oncology |
| Hainsworth et al. 2010^16^ | Adenocarcinoma of unknown primary | Breast oncology; Gastrointestinal oncology; Genitourinary oncology; Gynecologic oncology; Thoracic oncology |
| Pentheroudakis et al. 2008^17^ | Adenocarcinoma of unknown primary | Breast oncology; Gastrointestinal oncology; Genitourinary oncology; Gynecologic oncology; Thoracic oncology |
| Pouessel et al. 2004^18^ | Adenocarcinoma of unknown primary | Breast oncology; Gastrointestinal oncology; Genitourinary oncology; Gynecologic oncology; Thoracic oncology |
| Andersson et al. 2002^19^ | Allogeneic stem cell transplant^a^ | Lymphoid malignancy; Myeloid malignancy |
| Brunstein et al. 2011^20^ | Allogeneic stem cell transplant^a^ | Lymphoid malignancy; Myeloid malignancy |
| Kohrt et al. 2009^21^ | Allogeneic stem cell transplant^a^ | Lymphoid malignancy; Myeloid malignancy |
| Lowsky et al. 2005^22^ | Allogeneic stem cell transplant^a^ | Lymphoid malignancy; Myeloid malignancy |
| Maris et al. 2003^23^ | Allogeneic stem cell transplant^a^ | Lymphoid malignancy; Myeloid malignancy |
| Mohty et al. 2014^24^ | Allogeneic stem cell transplant^a^ | Lymphoid malignancy; Myeloid malignancy |
| Russell et al. 2002^25^ | Allogeneic stem cell transplant^a^ | Lymphoid malignancy; Myeloid malignancy |
| BRF117019^26^ | Basket trial^b^ | Endocrine oncology |
| MyPathway^27^ | Basket trial^b^ | Breast oncology; Gastrointestinal oncology; Genitourinary oncology; Gynecologic oncology; Head and Neck oncology; Thoracic oncology |
| VE-BASKET^28^ | Basket trial^b^ | Thoracic oncology; Gastrointestinal oncology; Plasma Cell Disorders; Myeloid malignancy; Endocrine oncology |
| MSK 15-335^29^ | Basket trial^b^ | Thoracic oncology |
| SUMMIT^30^ | Basket trial^b^ | Breast oncology; Gastrointestinal oncology; Genitourinary oncology; Gynecologic oncology; Thoracic oncology |
| KEYNOTE-016a^31^ | Basket trial^b^ | Gastrointestinal oncology |
| KEYNOTE-016b^32^ | Basket trial^b^ | Gastrointestinal oncology; Gynecologic oncology |
| LOXO-TRK-14001^33^ | Basket trial^b^ | Head and Neck oncology; Sarcoma; Endocrine oncology; Gastrointestinal oncology |

^a^Most allogeneic stem cell transplant trials on HemOnc.org are located on the disease-specific page(s) for which the treatment is intended. The trials here were nonrestrictive but in all cases patients with lymphoid and myeloid malignancies were evaluated.

^b^For basket trials, only those subtypes with at least 5 studied patients were included in the re-mapping. In the first KEYNOTE-016 publication (Le et al. 2015), only gastrointestinal oncology had at least 5 patients; in the second (Le et al. 2017), both gastrointestinal and gynecologic oncology had at least 5 patients.

# **Supplemental Bibliography**

1. Jarillo, J. C. On strategic networks. *Strategic Management Journal* **9**, 31–41 (1988).

2. Newman, M. E. J. Modularity and community structure in networks. *Proc Natl Acad Sci U S A* **103**, 8577–8582 (2006).

3. Newman, M. E. J. Assortative Mixing in Networks. *Phys. Rev. Lett.* **89**, 208701 (2002).

4. Freeman, L. C. A Set of Measures of Centrality Based on Betweenness. *Sociometry* **40**, 35–41 (1977).

5. Brin, S. & Page, L. The Anatomy of a Large-Scale Hypertextual Web Search Engine. in *Computer Networks and Isdn Systems* 107–117 (1998).

6. Martin, S. B., Brown, W. M., Klavans, R. & Boyack, K. W. DrL: Distributed Recursive (Graph) Layout. *Journal of Graph Algorithms and Applications* (2008).

7. Sinatra, R., Wang, D., Deville, P., Song, C. & Barabási, A.-L. Quantifying the evolution of individual scientific impact. *Science* **354**, aaf5239 (2016).

8. Briasoulis, E. *et al.* Carboplatin plus paclitaxel in unknown primary carcinoma: a phase II Hellenic Cooperative Oncology Group Study. *J. Clin. Oncol.* **18**, 3101–3107 (2000).

9. Culine, S. *et al.* Cisplatin in combination with either gemcitabine or irinotecan in carcinomas of unknown primary site: results of a randomized phase II study--trial for the French Study Group on Carcinomas of Unknown Primary (GEFCAPI 01). *J. Clin. Oncol.* **21**, 3479–3482 (2003).

10. Gross-Goupil, M. *et al.* Cisplatin alone or combined with gemcitabine in carcinomas of unknown primary: results of the randomised GEFCAPI 02 trial. *Eur. J. Cancer* **48**, 721–727 (2012).

11. Greco, F. A. *et al.* Carcinoma of unknown primary site: phase II trials with docetaxel plus cisplatin or carboplatin. *Ann. Oncol.* **11**, 211–215 (2000).

12. Greco, F. A. *et al.* Carcinoma of unknown primary site. *Cancer* **89**, 2655–2660 (2000).

13. Greco, F. A. *et al.* Gemcitabine, carboplatin, and paclitaxel for patients with carcinoma of unknown primary site: a Minnie Pearl Cancer Research Network study. *J. Clin. Oncol.* **20**, 1651–1656 (2002).

14. Hainsworth, J. D., Johnson, D. H. & Greco, F. A. Cisplatin-based combination chemotherapy in the treatment of poorly differentiated carcinoma and poorly differentiated adenocarcinoma of unknown primary site: results of a 12-year experience. *J. Clin. Oncol.* **10**, 912–922 (1992).

15. Hainsworth, J. D. *et al.* Phase II trial of bevacizumab and erlotinib in carcinomas of unknown primary site: the Minnie Pearl Cancer Research Network. *J. Clin. Oncol.* **25**, 1747–1752 (2007).

16. Hainsworth, J. D. *et al.* Paclitaxel/carboplatin/etoposide versus gemcitabine/irinotecan in the first-line treatment of patients with carcinoma of unknown primary site: a randomized, phase III Sarah Cannon Oncology Research Consortium Trial. *Cancer J* **16**, 70–75 (2010).

17. Pentheroudakis, G. *et al.* Docetaxel and carboplatin combination chemotherapy as outpatient palliative therapy in carcinoma of unknown primary: a multicentre Hellenic Cooperative Oncology Group phase II study. *Acta Oncol* **47**, 1148–1155 (2008).

18. Pouessel, D. *et al.* Gemcitabine and docetaxel as front-line chemotherapy in patients with carcinoma of an unknown primary site. *Cancer* **100**, 1257–1261 (2004).

19. Andersson, B. S. *et al.* Conditioning therapy with intravenous busulfan and cyclophosphamide (IV BuCy2) for hematologic malignancies prior to allogeneic stem cell transplantation: a phase II study. *Biol. Blood Marrow Transplant.* **8**, 145–154 (2002).

20. Brunstein, C. G. *et al.* Alternative donor transplantation after reduced intensity conditioning: results of parallel phase 2 trials using partially HLA-mismatched related bone marrow or unrelated double umbilical cord blood grafts. *Blood* **118**, 282–288 (2011).

21. Kohrt, H. E. *et al.* TLI and ATG conditioning with low risk of graft-versus-host disease retains antitumor reactions after allogeneic hematopoietic cell transplantation from related and unrelated donors. *Blood* **114**, 1099–1109 (2009).

22. Lowsky, R. *et al.* Protective conditioning for acute graft-versus-host disease. *N. Engl. J. Med.* **353**, 1321–1331 (2005).

23. Maris, M. B. *et al.* HLA-matched unrelated donor hematopoietic cell transplantation after nonmyeloablative conditioning for patients with hematologic malignancies. *Blood* **102**, 2021–2030 (2003).

24. Mohty, M. *et al.* Reduced-toxicity conditioning with fludarabine, once-daily intravenous busulfan, and antithymocyte globulins prior to allogeneic stem cell transplantation: results of a multicenter prospective phase 2 trial. *Cancer* **121**, 562–569 (2015).

25. Russell, J. A. *et al.* Once-daily intravenous busulfan given with fludarabine as conditioning for allogeneic stem cell transplantation: study of pharmacokinetics and early clinical outcomes. *Biol. Blood Marrow Transplant.* **8**, 468–476 (2002).

26. Subbiah, V. *et al.* Dabrafenib and Trametinib Treatment in Patients With Locally Advanced or Metastatic BRAF V600-Mutant Anaplastic Thyroid Cancer. *J. Clin. Oncol.* **36**, 7–13 (2018).

27. Hainsworth, J. D. *et al.* Targeted Therapy for Advanced Solid Tumors on the Basis of Molecular Profiles: Results From MyPathway, an Open-Label, Phase IIa Multiple Basket Study. *J. Clin. Oncol.* **36**, 536–542 (2018).

28. Hyman, D. M. *et al.* Vemurafenib in Multiple Nonmelanoma Cancers with BRAF V600 Mutations. *N. Engl. J. Med.* **373**, 726–736 (2015).

29. Li, B. T. *et al.* Ado-Trastuzumab Emtansine for Patients With HER2-Mutant Lung Cancers: Results From a Phase II Basket Trial. *J. Clin. Oncol.* **36**, 2532–2537 (2018).

30. Hyman, D. M. *et al.* HER kinase inhibition in patients with HER2- and HER3-mutant cancers. *Nature* **554**, 189–194 (2018).

31. Le, D. T. *et al.* PD-1 Blockade in Tumors with Mismatch-Repair Deficiency. *N. Engl. J. Med.* **372**, 2509–2520 (2015).

32. Le, D. T. *et al.* Mismatch repair deficiency predicts response of solid tumors to PD-1 blockade. *Science* **357**, 409–413 (2017).

33. Drilon, A. *et al.* Efficacy of Larotrectinib in TRK Fusion-Positive Cancers in Adults and Children. *N. Engl. J. Med.* **378**, 731–739 (2018).
